# Upregulated Long Non-coding RNA Lnc-MRPL39-2:1 Induces the Growth and Invasion of Nasopharyngeal Carcinoma by Binding to HuR and Stabilizing β-Catenin mRNA

**DOI:** 10.7150/ijbs.79115

**Published:** 2023-04-25

**Authors:** Yunhong Tian, Meiling Ai, Chunshan Liu, Yuchao Wu, Muhammad Khan, Baiyao Wang, Huidong Long, Chunyue Huang, Jie Lin, Anan Xu, Rong Li, Bohong Cen, Wenze Qiu, Guofeng Xie, Yawei Yuan

**Affiliations:** 1 Department of Radiation Oncology, Affiliated Cancer Hospital & Institute of Guangzhou Medical University, Guangzhou, Guangdong Province People's Republic of China; 2 State Key Laboratory of Respiratory Disease, Affiliated Cancer Hospital & Institute of Guangzhou Medical University, Guangzhou China

**Keywords:** lncRNAs lnc-MRPL39-2:1, nasopharyngeal carcinoma, HuR, β-catenin

## Abstract

Long non-coding RNAs (lncRNAs) have been to regulate tumor progression and therapy resistance through various molecular mechanisms. In this study, we investigated the role of lncRNAs in nasopharyngeal carcinoma (NPC) and the underlying mechanism. Using lncRNA arrays to analyze the lncRNA profiles of the NPC and para-tumor tissues, we detected the novel lnc-MRPL39-2:1, which was validated by *in situ* hybridization and by the 5' and 3' rapid amplification of the cDNA ends. Further, its role in NPC cell growth and metastasis was verified *in vitro* and *in vivo*. The researchers conducted the RNA pull-down assays, mass spectrometry (MS), dual-luciferase reporter assays, RNA immunoprecipitation (RIP) assays, and the MS2-RIP assays were then used to identify the lnc-MRPL39-2:1-interacting proteins and miRNAs. We found that lnc-MRPL39-2:1, which was highly expressed in in NPC tissues, was related to a poor prognosis in NPC patients. Furthermore, lnc-MRPL39-2:1 was shown to induce the growth and invasion of NPC by interacting directly with the Hu-antigen R (HuR) to upregulate β-catenin expression both* in vivo* and *in vitro*. Lnc-MRPL39-2:1 expression was also suppressed by microRNA (miR)-329. Thus, these findings indicate that lnc-MRPL39-2:1 is essential in NPC tumorigenesis and metastasis and highlight its potential as a prognostic marker and therapeutic target for NPC.

## Introduction

Nasopharyngeal carcinoma (NPC) is a type of head and neck cancer that originates in the nasopharynx and displays a high metastatic potential 1. It is characterized by its distinctive geographical distribution and primarily affects people in Southeast Asia and North Africa. In Southern China, the age-standardized incidence rate of NPC is 20 per 100,000 individuals 2, 3. Currently, a combination of chemotherapy and radiotherapy is used as the primary treatment strategy for NPC 4. However, since NPC is characterized by frequent recurrence and metastasis, the therapeutic effect of radiotherapy combined with chemotherapy is limited, and the prognosis is still poor 5. Hence, it is essential to explore the mechanism underlying NPC, find new therapeutic targets, and offer new treatment strategies.

Long non-coding RNAs (lncRNAs) are a group of endogenous transcripts that are longer than 200 nucleotides (nt) in length and are not translated into proteins 6. LncRNAs are important regulators of several biological processes, including cell growth, differentiation, metastasis, and cancer progression 7. Mechanistically, lncRNAs usually function as molecular signals, guides, scaffolds, and decoys to affect mRNA stability and translation 8. Several studies have demonstrated that dysregulated expression of lncRNAs is vital for cancer and that lncRNAs can promote or inhibit cancer progression 6. For example, lncRNA LINC02582 was shown to increase the radio-resistance of the breast cancer cells by acting as a competing endogenous RNA (ceRNA) that sponges the miR-200c 9. Moreover, several lncRNAs are associated with NPC. For example, lncRNA FAM225A was shown to promote NPC tumorigenesis and metastasis by increasing the ITGB3 expression level 10. Furthermore, lncRNA PVT1 increases the KAT2A acetyltransferase activity and stabilizes HIF-1α, thus promoting NPC cell growth 11. However, for the majority of the lncRNAs, their functions and molecular mechanisms of a majority of the lncRNAs in the regulation of NPC remain largely unknown. Therefore, in this study, we have investigated the lncRNA profiles of the NPC and para-tumor tissues and detected the novel lncRNA sequence, i.e., lnc-MRPL39-2:1. This lncRNA was expressed at high levels in the cytoplasm of NPC cells and played a vital role in the poor prognosis of advanced-stage NPC patients. Lnc-MRPL39-2:1 was also shown to induce the growth and invasion of NPC by interacting with the Hu-antigen R (HuR) to upregulate the β-catenin expression. Further experiments revealed that the lnc-MRPL39-2:1 expression was suppressed by the miR-329.

## Results

### Lnc-MRPL39-2:1 was upregulated in the NPC cells and was responsible for the poor prognosis in the NPC patients

**Figure 1:**
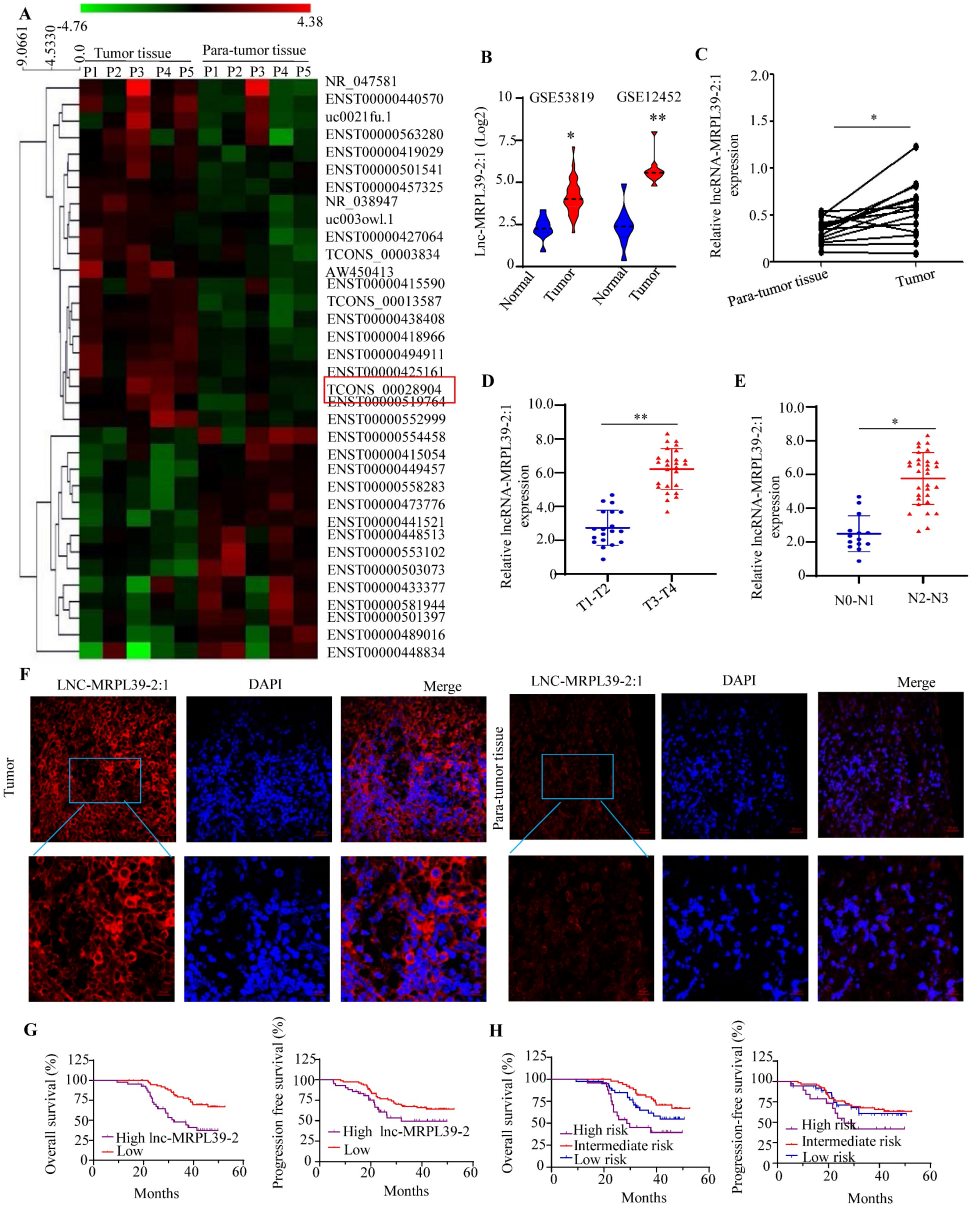
** Lnc-MRPL39-2:1 is expressed at a high level in NPC and is related to the poor survival of NPC patients.** (**A**) Heat map showing the top 35 differentially expressed lncRNAs between the NPC tumor and para-tumor tissues (n = 5). (**B**) LncRNA array data showing the upregulation of lnc-MRPL39-2:1 in NPC tumors from the GSE12452 and GSE53819 databases. (**C**) PCR analysis of lnc-MRPL39-2:1 expression as measured by qPCR in NPC and para-tumor tissues. (**D-E**) High lnc-MRPL39-2:1 expression in advanced T (**C**) and N stage (**D**) of NPC. (**F**) RNA-FISH analysis of lnc-MRPL39-2:1 expression in tumor and para-tumor tissues. (**G**) Kaplan-Meier analysis showing a negative correlation of the OS and PFS of NPC patients with lnc-MRPL39-2:1 levels. (**H**) High-risk groups are significantly involved with decreased duration of OS and PFS (*P < 0.05, **P < 0.01).

To determine the lncRNAs that play important roles in the NPC progression, we analyzed the expression profiles of a panel of lncRNA in the five selected pairs of NPC and para-tumor tissues using lncRNA arrays. The results indicated the presence of 262 dysregulated lncRNAs, a high proportion (48.15%) of which were intergenic (i.e., present in different chromosomes). The lncRNAs ranged from 400 bp to 800 bp in size with a range of classifications, and chromosomal locations (**Figs. S1**). The top 35 significantly-altered lncRNAs are shown in** Fig. 1A**. A few of the differentially expressed lncRNAs (four upregulated and three downregulated) were selected for further analysis (**Figs. S2A, B**). As one of the most highly upregulated lncRNAs detected, we focused primarily on lnc-MRPL39-2:1. To explore the role of the lnc-MRPL39-2:1 in NPC progression, we analyzed the differential expression of lnc-MRPL39-2:1 in the NPC tissues and the non-tumor tissues in the Gene Expression Omnibus database (GEO, https://www.ncbi.nlm.nih.gov/geo/). The results indicated that lnc-MRPL39-2:1 was expressed at a high level in NPC tumors compared to non-tumor tissues (**Fig. 1B**). Using quantitative PCR (qPCR) and the RNA-fluorescence *In Situ* Hybridization (RNA-FISH) techniques for validation, we confirmed lnc-MRPL39-2:1 was significantly upregulated in NPC tumor tissues compared with non-tumor tissues. Moreover, we established a direct link between lnc-MRPL39-2:1 expression levels and the advanced clinical stages of NPC patients (**Fig. 1C-F; Fig. S2C; Table S1**). ISH arrays were used to further identify the expression and localization of the lnc-MRPL39-2:1 in NPC. Lnc-MRPL39-2:1 expression was negligible in nasopharyngitis tissues, whereas upregulated lnc-MRPL39-2:1 expression was detected in 75.4% of the NPC samples (92/122) had a higher lnc-MRPL39-2:1 expression (**Fig. S4C**). The expression levels of the lnc-MRPL39-2:1 were dependent on many factors, including the NPC patients' tumor (T) stage, nodes (N) stage, and the tumor-node-metastasis (TNM) stage. However, it was not related to other pathological characteristics such as sex, age, and local treatment failure of the patients (**Table S1**). Survival analysis indicated that upregulated lnc-MRPL39-2:1 expression was associated with a poor overall survival (OS) rate and progression-free survival (PFS) (**Fig. 1G**). Multivariate analysis of the independent prognostic factors for OS and PFS indicated a negative correlation of lnc-MRPL39-2:1 expression and TNM stage with the prognostic factors for the OS and PFS (**Table S2**). Subsequent survival analysis based on a combination of the lnc-MRPL39-2:1 signature and TNM stage showed significant differences in the OS and PFS values amongst the low- (low lnc-MRPL39-2:1 expression and early TNM stage), intermediate- (high lnc-MRPL39-2:1 expression or advanced TNM stage), and high- (high lnc-MRPL39-2:1 expression and advanced TNM stage) risk groups, thereby indicating that a combination of the lnc-MRPL39-2:1 expression and TNM stage could act as a potential marker to predict the NPC prognosis (**Fig. 1H**). In conclusion, the data revealed that lnc-MRPL39-2:1 expression was increased in NPC and was related to the poor prognosis.

### Lnc-MRPL39-2:1 was identified as a novel lncRNA primarily expressed in the cytoplasm of the NPC cells

**Figure 2:**
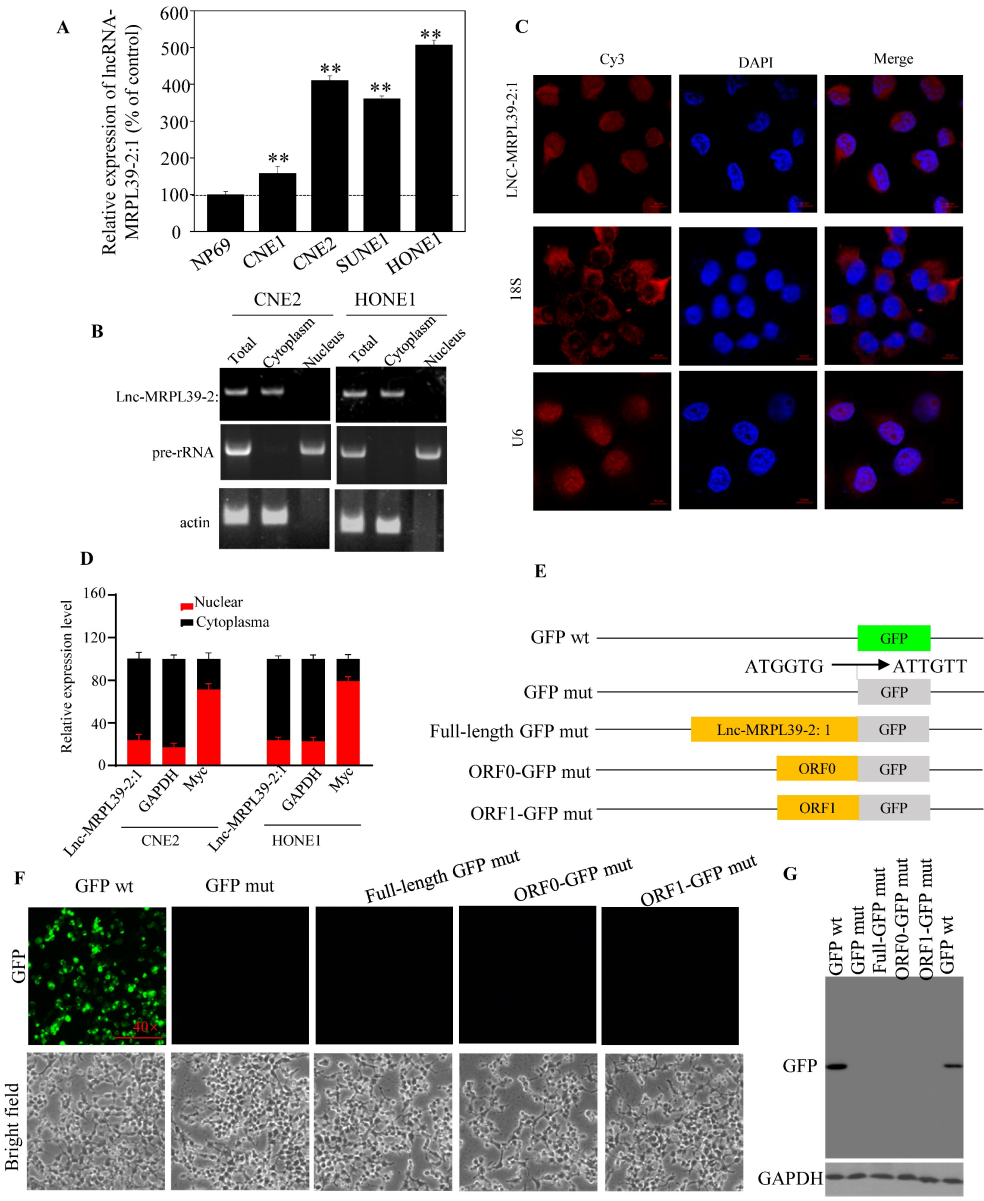
** Lnc-MRPL39-2:1, as a lncRNA, is primarily expressed in the cytoplasm of NPC cells.** (**A**) qPCR analysis of lnc-MRPL39-2:1 expression in NPC cell lines. (**B**) Agarose gel electrophoresis showing that lnc-MRPL39-2:1 is located mainly in the cytoplasm. (**C**) RNA-FISH analysis of the subcellular localization of lnc-MRPL39-2:1 in CNE2 cells. (**D**) qPCR analysis of lnc-MRPL39-2:1 expression levels after fractionation of CNE2 and HONE1 cells; MYC and GAPDH represent positive controls for the nuclear and cytoplasmic fractions, respectively. (**E-G**) Schematic diagram of the various lnc-MRPL39-2:1 constructs fused to GFP. The start codon ATGGTG of the wild type GFP (GFP wt) gene was mutated to ATTGTT (GFP mut). (**E**) HEK293T cells transfected with the various vectors were validated by microscopic evaluation (**F**) and western blot analysis (**G**).

Analysis of different cell lines showed higher expression of lnc-MRPL39-2:1 expression in different cell lines compared to that in a non-cancerous human nasopharyngeal epithelial cell line (NP69) (**Fig. 2A**). Furthermore, lnc-MRPL39-2:1 expression was primarily localized in the cytoplasm of the NPC cells (**Fig. 2B-D**). The complete lnc-MRPL39-2:1 sequence was then determined by the 5' and 3' cDNA ends (RACE) assay (**Fig. S5**). Searches of the phylogenetic codon substitution frequency (PhyloCSF), CPAT, and LNCipedia databases, confirmed the absence of protein-coding potential; however, RegRNA 2.0 analysis revealed that the lnc-MRPL39-2:1 transcript contains two open reading frames (ORFs). To determine translation potential of these two lnc-MRPL39-2:1 ORFs, we constructed vectors carrying a mutant initiation codon of the green fluorescent protein (GFP) that was fused downstream of the full lnc-MRPL39-2:1 transcript or potential ORFs (**Fig. 2E**). GFP expression was observed in cells transfected with a wild-type GFP overexpression vector, but not in cells transfected with a mutant construct (**Fig. 2F**). Furthermore, western blot analysis confirmed that the lnc-MRPL39-2:1 ORFs were not successfully translated into proteins (**Fig. 2G**). Thus, we concluded that the lnc-MRPL39-2:1 has no protein-coding ability and is detected primarily in the cytoplasm of NPC cells.

### Lnc-MRPL39-2:1 promoted NPC cell proliferation and migration *in vitro*

**Figure 3:**
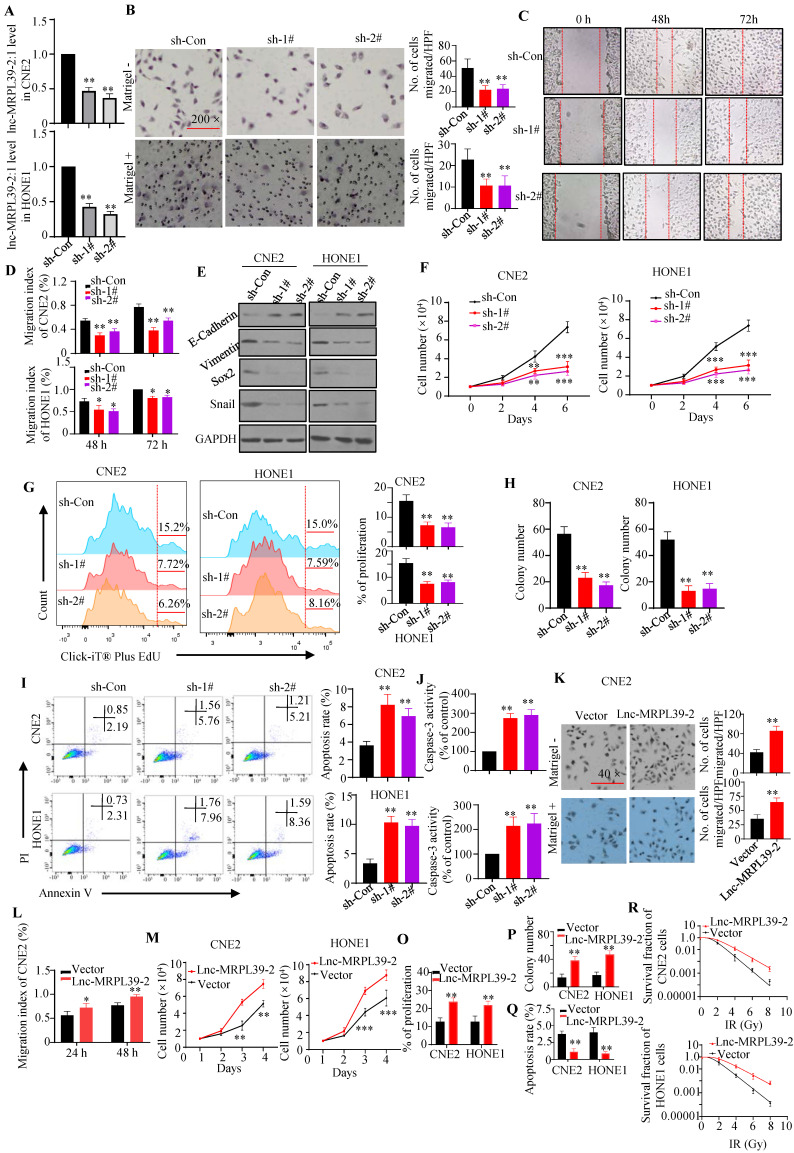
** Lnc-MRPL39-2:1 promotes NPC cell proliferation and invasion.** (**A**) Lnc-MRPL39-2:1 expression is stably downregulated in the NPC cell lines transfection with sh#1 and sh#2. (**B**-**E**) Downregulation of lnc-MRPL39-2:1 inhibits CNE2 cell migration and invasion. (**B**) Transwell migration and invasion assays of the effect of lnc-MRPL39-2:1 on the migration and invasion of NPC cells. (**C** and** D**) Wound-healing assays showing the increased invasion ability of NPC cells induced by lnc-MRPL39-2:1; cells were stained using 0.1% crystal violet. (**E**) Epithelial-to- mesenchymal transition markers in NPC cells. (**F**-**J**) Lnc-MRPL39-2:1 knockdown significantly inhibits NPC cell growth. (**F**) Effects of lnc-MRPL39-2:1 knockdown on NPC proliferation as measured by the MTS (**F**) and Click-iT^®^ Plus EdU (**G**) assays, colony-forming ability (**H**), apoptosis measured by flow cytometry (**I**) and caspase-3 activity (**J**). Lnc-MRPL39-2:1 overexpression-induced enhanced migration as measured by transwell (**K**) and wound-healing (**L**) assays. Measurement of proliferation (**M** and **O**) and colony-forming ability (**P**) of NPC cells. (**Q**) Lnc-MRPL39-2:1 overexpression-induced enhanced apoptosis measured by flow cytometry. (**R**) Colony‐forming ability of CNE2 and HONE1 cells exposed to various doses of irradiation. The survival fraction (SF) assay was analyzed using the single‐hit multi‐target theory formula…The experiments were replicated more than three times separately (^*^P < 0.05, ^**^P < 0.01).

To validate the roles of lnc-MRPL39-2:1 in NPC cells, stable lnc-MRPL39-2:1-knockdown CNE2 and HONE1 cell lines were established (**Fig. 3A**). Lnc-MRPL39-2:1 knockdown significantly inhibited NPC cell migration and invasion (**Fig. 3B-E**; **Fig. S4A-B**). Furthermore, the researchers conducted the MTS and EdU assays revealed that the NPC cell proliferation was significantly attenuated following lnc-MRPL39-2:1 knockdown (**Fig. 3F**, **G**). Colony-forming assays showed that lnc-MRPL39-2:1 knockdown decreased colony-formation in the NPC cells (**Fig.3H**; **Fig. S4C**). Moreover, lnc-MRPL39-2:1 knockdown significantly increased NPC cell apoptosis (**Fig. 3I**, **J**). Overexpression of lnc-MRPL39-2:1 in transfected in NPC cells was confirmed by qPCR analysis (**Fig. S4D**). Lnc-MRPL39-2:1 overexpression markedly enhanced cell invasion, proliferation, and the clonogenic capacity of NPC cells (**Fig. 3K-P**; **Fig. S4E-F**). However, its overexpression decreased the number of apoptotic NPC cells and enhanced the radio-resistance of NPC cells (**Fig. 3Q-R**; **Fig. S4G**). In addition, lnc-MRPL39-2:1 overexpression promoted cell invasion, proliferation, and the colony-formation, and decreased apoptosis of the human immortalized nasopharyngeal epithelial cell line NP69 *in vitro* (**Fig. S5**)*.* In conclusion, these results showed that lnc-MRPL39-2:1 increased the proliferative and metastatic capacity of NPC cells* in intro*.

### Lnc-MRPL39-2:1 enabled NPC cell growth and metastasis by interacting directly with HuR

**Figure 4:**
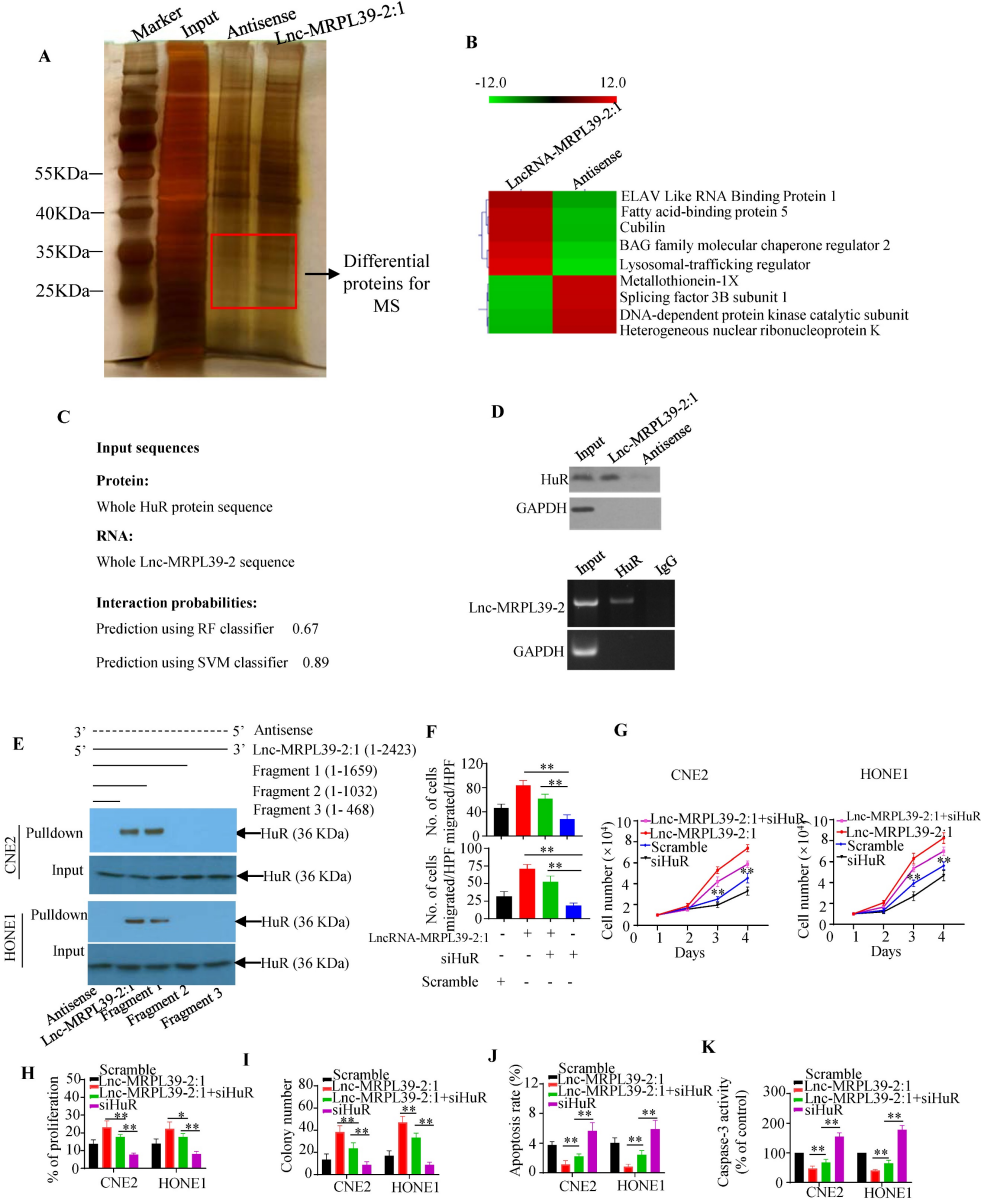
** HuR is responsible for lnc-MRPL39-2:1-mediated cell growth, migration, and invasion.** (**A**) Silver-stained RNA pull-down gel showing biotin-labeled lnc-MRPL39-2:1 incubated with NPC cell lysates and samples used for mass spectrometry. (**B**) Heat map showing he top five upregulated and top four downregulated proteins determined by MS analysis. (**C**) Direct interaction between lnc-MRPL39-2:1 and HuR as predicted by the online tool, RPISeq. (**D**) Interaction between lnc-MRPL39-2:1 and HuR confirmed by western blot and RIP assays using anti-HuR antibody and specific probes to detect lnc-MRPL39-2:1. (**E**) Interaction of different different fragments of lnc-MRPL39-2:1 or its antisense sequence. Their interaction with HuR was validated by RNA pulldown and western blot assays. (**F-K**) CNE2 or HONE1 cells transfected with lnc-MRPL39-2:1-overexpression vector and/or siHuR vector. Cell migration was determined by the transwell assay (**F**). Cell proliferation was validated by MTS (**G**) and EdU (**H**) assays. (**I**) NPC cell colony-formation assay. NPC cell apoptosis measured by flow cytometry (**J**) and caspase-3 activity (**K**). Data are expressed as mean ± SEM of triplicate assays (*P < 0.05, **P < 0.01).

To identify the proteins interacting with lnc-MRPL39-2:1 in NPC cells, we performed RNA pulldown assay with biotin-labeled lncRNAs (**Fig. 4A**). Lnc-MRPL39-2:1 was shown to bond directly to a 25-40 kD protein. Approximately 130 proteins were detected by MS analysis of the corresponding band, including the RNA binding protein (RBP) HuR, which is involved in the post-transcriptional regulation of cancer-related gene expression and interacts directly with lnc-MRPL39-2:1 (**Fig. 4B**). Immunohistochemical (IHC) analysis showed a higher level of HuR in NPC tissues than in para-tumor tissues. Moreover, patients with a high HuR expression had a shorter OS and PFS compared to those with low HuR expression (**Fig. S6A, B**). Next, lnc-MRPL39-2:1 was predicted to bond directly to the HuR protein by the RPISeq software. The direct interaction between lnc-MRPL39-2:1 and HuR was further verified by western blot of the lnc-MRPL39-2:1 pulldown cell lysate using an anti-HuR antibody and RNA immunoprecipitation (RIP) assay (**Fig. ​4C, D**). Furthermore, to determine the HuR binding site within lnc-MRPL39-2:1, different deletion fragments were constructed based on their secondary structure as predicted by the RNA fold Web server. RNA pull-down and western blot assays indicated that a 1032-1659 nt region of lnc-MRPL39-2:1 was required for the interaction between lnc-MRPL39-2:1 and HuR (**Fig. ​4E**). Finally, to determine the role of HuR in the tumor-promoting effects of lnc-MRPL39-2:1, CNE2 and HONE1 cells were transfected with lnc-MRPL39-2:1-overexpression vector and si-HuR. HuR knockdown partially abolished the lnc-MRPL39-2:1-induced proliferation and metastasis of NPC cells (**Fig. 4F**-**I**; **Fig. S6C, D**). Furthermore, the lnc-MRPL39-2:1-mediated inhibition of NPC cell apoptosis was rescued by si-HuR (**Fig. ​4J, K**; **Fig. S6E**). Thus, we concluded that the lnc-MRPL39-2:1 promoted NPC cell growth and metastasis by directly binding to the HuR.

### Lnc-MRPL39-2:1 promoted cell growth and metastasis by enhancing β-catenin expression

**Figure 5:**
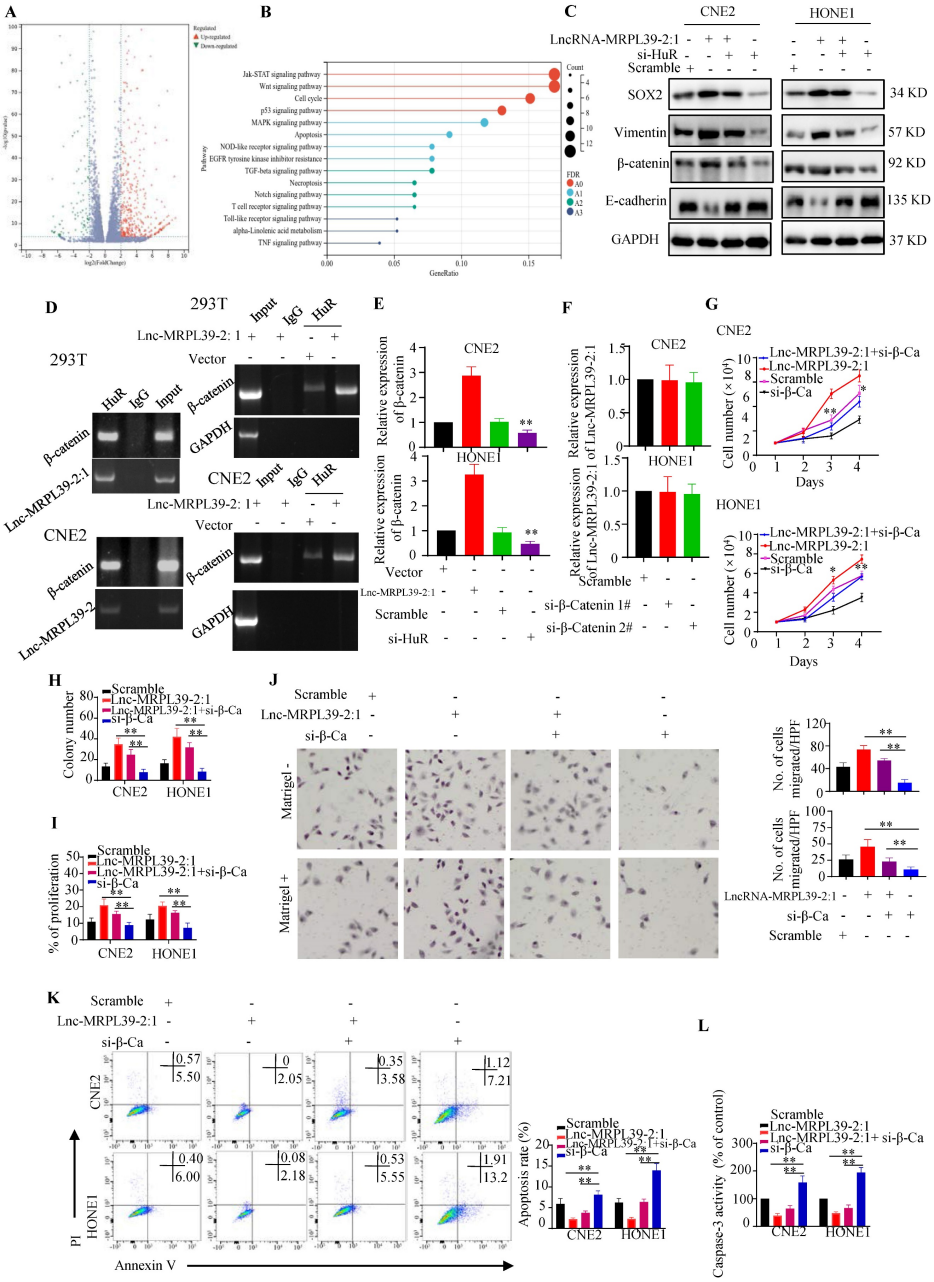
** Lnc-MRPL39-2:1 promotes NPC cell growth and metastasis by enhancing β-catenin expression.** (**A**) Volcano plots showing differentially expressed mRNAs in a lnc-MRPL39-2:1 overexpressing cell line versus the vector group. (**B**) Bubble plot of KEGG enrichment analysis of differentially expressed mRNAs. (**C**) Western blot analysis of the expression of β-catenin signaling pathway proteins and EMT marker proteins. (**D**) Interaction between β-catenin and HuR confirmed by RIP assay using the anti-HuR antibody in HEK293T and CNE2 cell lines. (**E**) qPCR analysis of β-catenin mRNA expression in CNE2 (upper panel) and HONE1 (lower panel) cells transfected with lnc-MRPL39-2:1 or si-HuR. (**F**) qPCR validation of lnc-MRPL39-2:1 expression in NPC cells transfected with si-β-catenin. (**G-I**) Effects of lnc-MRPL39-2:1 and β-catenin on NPC cell proliferation measured by MTS (**G**), colony formation (**H**), and Click-iT^®^ Plus EdU (**I**) assays. (**J**) Migration of CNE2 or HONE1 cells transfected with lnc-MRPL39-2:1-overexpression vector and/or si-β-catenin vector measured by transwell assay. (**K, L**) Apoptosis of cells transfected with lnc-MRPL39-2:1-overexpression vector and/or si-β-catenin vector measured by flow cytometry (**K**) and caspase-3 activity (**L**) (^*^P < 0.05, ^**^P < 0.01).

Next, we performed RNA sequencing to determine downstream targets potentially involved in the mechanism by which lnc-MRPL39-2:1 promoted NPC cell growth and metastasis. Our analysis revealed 890 significantly up-regulated and 247 down-regulated mRNAs (≥2.0-fold, *P* < 0.05) in lnc-MRPL39-2:1 overexpressed CNE2 cells compared to the control group (**Fig. 5A**). KEGG analysis indicated that these differentially expressed genes were enriched in the JAK-STAT signaling pathway, Wnt signaling pathway, cell cycle, and the P53 and MAPK signaling pathway (**Fig. 5B**). These results revealed that lnc-MRPL39-2:1 may be a vital regulatory lncRNA involved in regulating NPC cell progression. Beta-catenin was one of the most upregulated factors. Previous studies confirmed that HuR can interact directly with the β-catenin mRNA to enhance both its half-life and expression 12, 13. In this study, western blot analysis indicated that β-catenin, vimentin, β-catenin and SOX2 were upregulated by lnc-MRPL39-2:1 and down-regulated by HuR knock down (Fig. 5C). Thus, we hypothesized that lnc-MRPL39-2:1 stabilizes the β-catenin mRNA by enhancing the interaction between the HuR and β-catenin mRNA. To test this hypothesis, we first measured HuR and β-catenin expression at the mRNA and protein levels in cancer tissue. The expression of β-catenin correlated positively with that of HuR in NPC tissue (**Fig. S7A, B**). Next, we performed RIP assays to examine the role of lnc-MRPL39-2:1 in the interactions between HuR and β-catenin mRNA. Lnc-MRPL39-2:1 was shown to interact directly with HuR and enhanced the interaction between the HuR and β-catenin mRNAs (**Fig. 5D**). Lnc-MRPL39-2:1 overexpression increased the levels of beta catenin. However, the lnc-MRPL39-2:1 expression was not decreased by β-catenin knockdown, indicating that β-catenin is a downstream effector of lnc-MRPL39-2:1 (**Fig. 5E, F**). Moreover, β-catenin silencing abrogated the ability of lnc-MRPL39-2:1 to promote NPC cell proliferation and metastasis in NPC cells (**Fig. 5G-J**). Furthermore, β-catenin knockdown increased NPC cell apoptosis (**Fig. 5K, L**). In conclusion, these results indicated that β-catenin is required in the mechanism by which lnc-MRPL39-2:1 promotes NPC proliferation and metastasis.

### Lnc-MRPL39-2:1 was a direct target of miR-329

**Figure 6:**
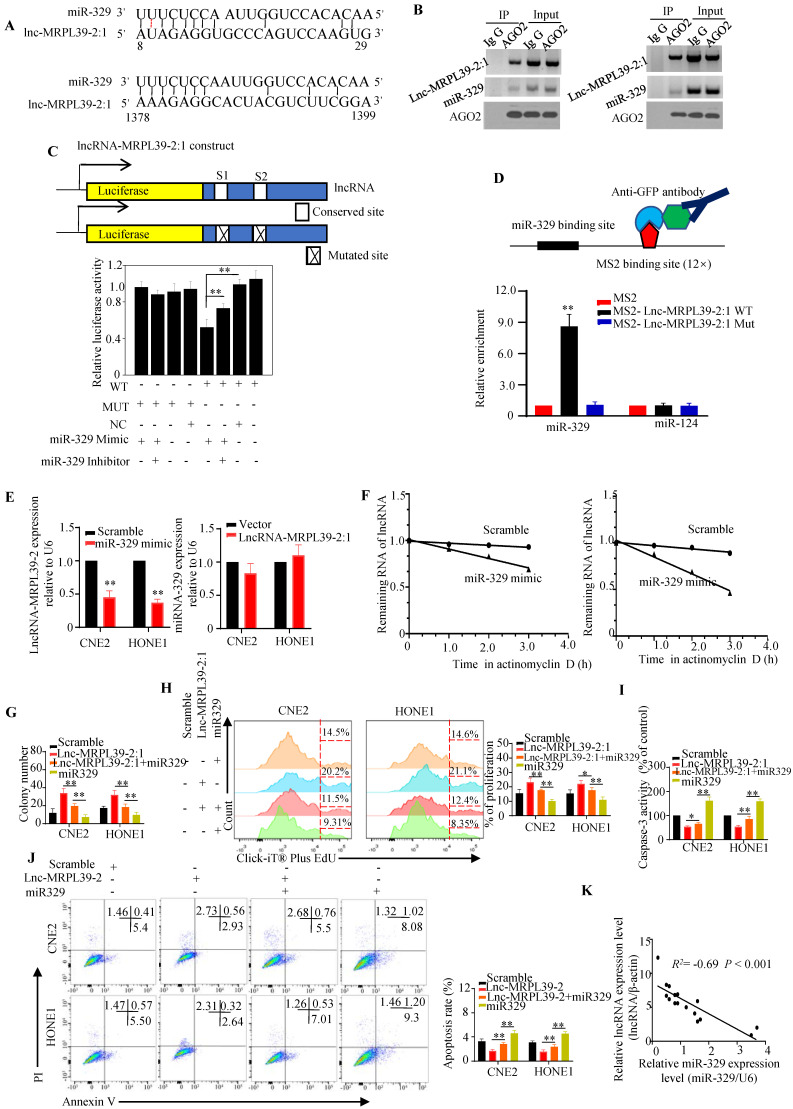
** Lnc-MRPL39-2:1 interacts directly with miR-329.** (**A**) Predicted miR-329 binding sites in the lnc-MRPL39-2:1 transcript. (**B**) Interaction of lnc-MRPL39-2:1 with miR-329 was validated by immunoprecipitation with the anti-Ago2 antibody or IgG (negative control) and measured by western blot. (**C**) Dual luciferase assay of HEK293T cells co-transfected with miR-329 and vectors carrying the wild-type or mutant lnc-MRPL39-2:1 sequence. (**D**) MS2-RIP assay verification of the interaction between lnc-MRPL39-2:1 and miR-329; miR-124 serves as a negative control. (**E**) Left: Decreased lnc-MRPL39-2:1 expression in CNE2 and HONE1 cells after the transfection with miR-329. Right: Expression of miR-329 in CNE2 and HONE1 cells overexpressing lnc-MRPL39-2:1 overexpression. (**F**) Reduction in the half-life of lnc-MRPL39-2:1 in miR-329-transfected CNE2 (left) and HONE1 cells (right). (**G-H**) MiR-329 abolished the increased colony formation (**G**) and proliferation ability (**H**) induced by lnc-MRPL39-2:1. (**I-J**) The decreased rate of apoptosis induced by lnc-MRPL39-2:1 is partly rescued by miR-329 measured by caspase-3 activity (**I**) and flow cytometry (**J**). (**K**) Scatter diagram exhibiting a negative correlation of lnc-MRPL39-2:1 and miR-329 in NPC tissues (^*^P < 0.05, ^**^P < 0.01).

Cytoplasmic lncRNAs function as ceRNAs by effectively binding to specific miRNAs and act as ceRNAs 14 15. Thus, we hypothesized that lnc-MRPL39-2:1 might promote NPC cell proliferation and metastasis by interacting with miRNA. Using the human lncRNA database LNCipedia (https://lncipedia.org/), we identified several miRNAs predicted to interact directly with lnc-MRPL39-2:1 (**Table S3**). Moreover, results from bioinformatic analysis predicted that miR-329 might bind to lnc-MRPL39-2:1 at two putative complementary sequences (**Fig. 6A**). RIP experiments showed that both lnc-MRPL39-2:1 and miR-329 were enriched in the AGO2 antibody-associated complex, indicating that miR-329 could directly interacts directly lnc-MRPL39-2:1 (**Fig. ​6B**). Moreover, we found that miR-329 decreased the activity of the luciferase activity in cells transfected with the wild-type lnc-MRPL39-2:1 construct (**Fig. 6C**), and the MS2-RIP assay indicated that miR-329, but not the negative control miR-124, bound to the lnc-MRPL39-2:1 at the targeting site (**Fig. ​6D**).

Further experiments to determine the regulatory relationship between lnc-MRPL39-2:1 and miR-329 showed that miR-329 suppressed lnc-MRPL39-2:1 expression, whereas lnc-MRPL39-2:1 overexpression did not affect miR-329 expression, indicating that lnc-MRPL39-2:1 is targeted by miR-329 (**Fig. ​6E**). Moreover, miR-329 overexpression shortened the lnc-MRPL39-2:1 half-life in CNE2 and HONE1 cells (**Fig. 6F**). Furthermore, we demonstrated the involvement of miR-329 in the mechanism by which lnc-MRPL39-2:1 promoted NPC cell growth and metastasis by co-transfecting NPC cell lines with an lnc-MRPL39-2:1 plasmid with or without miR-329 mimics. MiR-329 overexpression inhibited the ability of lnc-MRPL39-2:1 to promote tumor growth and invasion (**Fig. ​6G-J**). The negative correlation between the lnc-MRPL39-2:1 and miR-329 expression in NPC tissues was confirmed by qPCR (**Fig. ​6K**). Thus, the carcinogenic effect of lnc-MRPL39-2:1 was shown to be partially mediated by the negative regulation of miR-329.

### Lnc-MRPL39-2:1 promoted NPC progression *in vivo*

**Figure 7:**
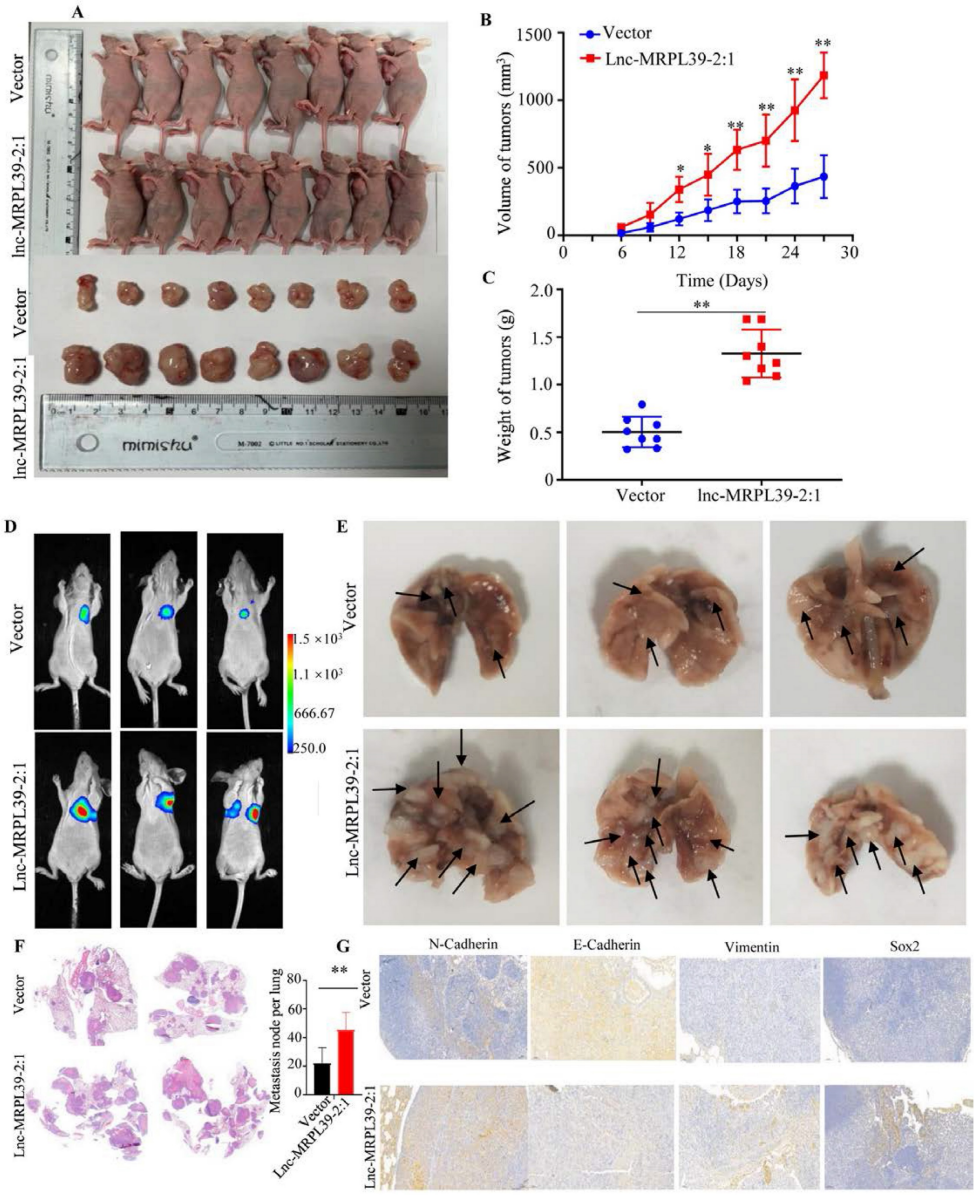
** Lnc-MRPL39-2:1 promoted NPC cell proliferation and metastasis *in vivo:*** (**A**) Images of the mice and tumors in the vector only and lnc-MRPL39-2:1 groups. (**B**) Weight of subcutaneous tumors. (**C**) Tumor volumes. (**D**) Bioluminescence images of mice bearing NPC xenografts transfected with lnc-MRPL39-2:1 or only vector only. (**E**) Images of lung lesions following injection of NPC cells transfected with lnc-MRPL39-2:1 or vector only. Metastatic lesions are indicated by arrowheads. (**F**) Representative image of lung with metastasis validated by hematoxylin and eosin staining. (**G**) The total number of metastatic lesions in mouse lungs; data represent the mean ± SD (*P < 0.05, **P < 0.01).

Having observed the pivotal role of lnc-MRPL39-2:1 in promoting NPC growth and invasion *in vitro*, we then confirmed its role in NPC progression *in vivo* in a nide mouse tumor xenograft model. We observed a remarkable elevation in the final tumor weight and volume of tumors in the lnc-MRPL39-2:1 overexpression group compared to the control group, indicating that lnc-MRPL39-2:1 significantly promoted tumor growth (**Fig. 7A-C**). We also examined the *in vivo* effects of lnc-MRPL39-2:1 overexpression on cell proliferation by carrying out IHC and hematoxylin and eosin (H&E) staining. Tumor cells invasion was observed in the epidermal appendages in the lnc-MRPL39-2:1 group but not in the control group. The relative ratio of Ki67-expressing cells was also higher in the lnc-MRPL39-2:1 group compared to that in the vector only group (**Fig. S8**). Finally, in the lung metastasis model, we detected a higher total number of metastatic lesions in the lnc-MRPL39-2:1 overexpression group compared with that in the control group (**Fig. 7D-G**). Thus, our findings indicate that lnc-MRPL39-2:1 contributes to NPC tumorigenesis and metastasis *in vivo*.

## Discussion

To improve the poor prognosis of patients with NPC, it is necessary to decipher the molecular mechanisms involved in its recurrence and metastasis. Various genomic and epigenomic mechanisms are being investigated, with lncRNAs considered to play crucial roles in cancer proliferation and metastasis. In this study, we explored the roles of lncRNAs in NPC and the underlying mechanism. We identified a novel oncogenic lncRNA, lnc-MRPL39-2:1 that was upregulated in NPC tissues and associated with advanced tumor stage and poor prognosis of patients. Mechanistically, lnc-MRPL39-2:1 induced the growth and invasion of NPC by interacting with HuR to upregulate β-catenin expression, both *in vivo* and* in vitro*. Additionally, lnc-MRPL39-2:1 expression could be suppressed by miR-329.

To date, tens of thousands of lncRNAs have been identified and they were found to play crucial roles in different types of cancers. Moreover, several studies have demonstrated that dysregulated lncRNAs regulate NPC progression 16. For instance, 10 lncRNA FAM225A, which is highly expressed in NPC patients, is associated with shorter OS since by promoting the invasion and proliferation of the NPC cells. Mechanistically, lncRNA FAM225A increases the mRNA levels of ITGB3 through competitively binding miR-590-3p and miR-1275, eventually promoting NPC cell growth and migration. LncRNA RP11-624L4.1 and TINCR are related to shorter survival by promoting NPC cell growth 17 18 Moreover, 11 lncRNA PVT1, which is expressed at a high levels in the NPC tissue, is associated with the poor prognosis of patients by promoting NPC cell growth and colony-formation. Similarly, in the current study, we found that lnc-MRPL39-2:1, which was upregulated in NPC tissues, was positively correlated with late tumor stage and poor survival of patients, thus indicating that lnc-MRPL39-2:1 may be a vital prognostic factor in NPC.

Nuclear lncRNAs and their functions have been studied quite extensively. However, cytoplasmic lncRNAs, which are not well understood, can also regulate protein localization and turnover and mRNA translation and stability. Some studies have shown that lncRNAs promoted the invasion and progression capacities of the NPC cells by regulating some critical targets, such as ITGB3, CDK4/6, or KAT2A acetyltransferase 10, 11, 17. In this study, we demonstrated that lnc-MRPL39-2:1, was expressed mainly in the cytoplasm of the NPC cells, and bound directly bind to HuR to elevate the β-catenin expression level 13. In addition, the interaction between lnc-MRPL39-2:1 and β-catenin mRNA was verified in NPC cells, further suggesting that lnc-MRPL39-2:1 plays a vital role in stabilizing β-catenin. Moreover, we identified a region between 1032-1659 nts of lnc-MRPL39-2:1 that was required for its interaction with HuR. These results indicate that lnc-MRPL39-2:1 exerts its effects on NPC cells by binding to HuR.

HuR participates in the post-transcriptional regulation of several cancer-associated genes associated with cell proliferation and metastasis. For instance, HuR stabilized snail mRNA by binding to the downstream factor of the mRNA, thus promoting metastasis of pancreatic cancer cells 19. Cai* et al.* reported that HuR increased colorectal cancer growth and oxaliplatin resistance by regulating CDC6 20. Moreover, Cao* et al.* showed that lincRNA-UFC1 binds HuR leading to upregulation of β-catenin expression at both the mRNA and protein levels^13^. Hence, in accordance with these previous findings, we showed that HuR is upregulated in human NPC tissues, promotes cell growth and metastasis, and is related to poor OS and PFS in NPC patients.

Emerging data have indicated that lncRNAs function as the miRNA sponges. For instance, 21 lncRNA-HGBC promotes gallbladder cancer proliferation and invasion by sponging miR-502-3p. In breast cancer, SNORD3A sensitizes cells to chemotherapy by acting as a miRNA sponge for miR-185-5p, leading to uridine monophosphate synthetase protein upregulation 15. Moreover, 14 lncRNA THAP9-AS1 is directly related to the poor survival rate of the patients with pancreatic ductal adenocarcinoma by sponging miR-484. Similarly, in this study, lnc-MRPL39-2:1 was identified as a molecular sponge of miR-329. In addition, dual-luciferase reporter assays confirmed that miR-329 interacted with lnc-MRPL39-2:1, as shown by the dual-luciferase reporter assay, and the expression of lnc-MRPL39-2:1 was downregulated by miR-329. Thus, these findings demonstrate that lnc-MRPL39-2:1 induces the growth and invasion of NPC by sponging the tumor-inhibiting miR-329.

To conclude, the results of this study demonstrated that lnc-MRPL39-2:1 is upregulated in the NPC tissues and accurately predicts a poor survival rate of NPC patients in an accurate manner. Furthermore, it was shown that the lncRNA attenuates the growth and invasion of NPC by interacting with HuR, leading to upregulation of β-catenin expression both* in vivo* and *in vitro*. These findings indicate that lnc-MRPL39-2:1 is essential in NPC tumorigenesis and metastasis and highlighted its potential as a prognostic marker and therapeutic target for NPC. Although, our findings provide insights into the value of lnc-MRPL39-2:1 as a promising therapeutic target against NPC, further studies are required to develop precise strategies targeting lnc-MRPL39-2:1 signaling and clarify the role of lnc-MRPL39-2:1 in other cancers.

## Materials and Methods

### Collection of biopsy specimens

Freshly frozen NPC and adjacent tissue samples were acquired from the Affiliated Cancer Hospital & Institute of Guangzhou Medical University. To analyze disease prognosis, we examined 121 NPC cases between January 2008 and December 2010 at the Affiliated Cancer Hospital & Institute of Guangzhou Medical University. All NPC patients were being treated using a standard treatment protocol. The patients had signed a written and informed consent form before being enrolled in the study. Thereafter, the researchers approached the Affiliated Cancer Hospital & Institute of Guangzhou Medical University and obtained approval for the study from its Institutional Ethical Review Board.

### Microarray analysis

Differentially expressed lncRNAs in NPC tissue were measured with the help of the Arraystar Human LncRNA Array V3.0 (KangChen Bio-tech, Shanghai, China). Briefly, total mRNA purified from the NPC tumor and para-tumor tissues was quantified using the NanoDrop ND-1000 technique. Then, mRNA was amplified and labeled according to the kit manufacturer's protocol (Nimblegen Systems, Inc., Madison, WI, USA). Then, the researchers purified the labeled RNAs and determined their quality. Then, they hybridized it onto the Human LncRNA Array v3.0. After washing the slides, they were scanned by a DNA microarray scanner (G2505C Series, Agilent Technologies, USA). Finally, the differentially expressed lncRNAs and mRNAs between the tumor and para-tumor tissues were validated according to fold change filtering. Further, the researchers carried out the KO and GO analyses to investigate the role played by the lncRNAs in cancer.

### Cell lines

Four NPC cell lines, i.e., CNE1, CNE2, SUNE1, and HONE1, along with the human immortalized nasopharyngeal epithelial cell line NP69 were obtained from the Shanghai Institute of Cell Biology (Shanghai, China). Every new batch of cell lines underwent mycoplasma testing and analysis of short tandem repeats. All the cell lines were incubated in the RPMI 1640 medium (Invitrogen, Thermo Fisher Scientific, Inc., Waltham, MA, USA) that contained 10% Fetal Bovine Serum (FBS, Gibco, Australia), penicillin, and streptomycin (100 U/mL, each) at the optimal temperature of 37 °C and with 5% CO_2_.

### RIP assay

The researchers performed the RIP assay using the Magna RIP RNA-Binding Protein Immunoprecipitation Kit (Millipore, MA, USA). Briefly, the cells that were transfected with or without a vector were lysed with lysis buffer (20 mM HEPES, 1 mM DTT, 0.1 M NaCl, 1 mM EDTA, 0.5% (v/v) Triton X-100, and 10% (v/v) glycerol) that also contained 1 mM proteinase and phosphatase inhibitor cocktail (Sigma-Aldrich, Missouri, USA), and the RNase inhibitor (Sigma-Aldrich, USA). Then, the primary antibodies or IgG (Cell Signaling Technology, Massachusetts, USA) were introduced into the cell lysates and this mixture was incubated overnight at 4 °C. Magnetic beads (Invitrogen) that were washed with the immunoprecipitation lysis buffer were added to the cell lysate mixture and incubated for 1 h at 4 °C. Finally, the beads were harvested and incubated at 70 °C for reversing the crosslinks. The sample was centrifuged and the RNAs in the supernatant were validated by qPCR. The results are presented as a fold change.

### RACE assay

Total RNA was isolated from the CNE2 cells using TRIzol (Invitrogen) and processed following the Trizell manufacturer's protocol. Subsequently, the researchers synthesized the cDNA from the extracted RNA using a cDNA Amplification kit (Bio-Rad, Hercules, CA, USA). Then, they carried out the 5′ RACE and 3′ RACE reactions using a 5′ Full RACE Kit (Takara, Dalian, China) and 3′ Full RACE Core Set with PrimeScript RTase Kit (Takara, China), respectively. Finally, the researchers separated the PCR products by electrophoresing the samples on a 1.5% agarose gel and then verified the products based on sequence analysis.

### RNA-ISH assay

The expression of lnc-MRPL39-2:1 was measured in paraffin-embedded samples using an ISH kit (Roche, IN, USA) based on the manufacturer's protocol. For this assay, the researchers cut the paraffin-embedded tissues into 4 μm thick sections. Then, these slides were deparaffinized and deproteinated. The specimens were incubated in the prehybridization solution at 42°C for 2 h. They were then incubated with a Digoxin-labeled oligonucleotide solution (Exiqon, USA) complementary to lnc-MRPL39-2:1 at 37 °C overnight. Next, these slides were washed and stained with 3,3′-Diaminobenzidine (Sigma-Aldrich) and hematoxylin solutions. Finally, the slides were imaged, and the stained loci were counted under a microscope (Nikon, Tokyo, Japan). The probe sequences for lnc-MRPL39-2:1 were as follows: AGAGGTGCCCAGTCCAAGTGAAGGGCAAGAGTCTACATAC; AACTATTACTCTAAAATGAAAGAATTCCTTTAAATTGAAT; GTCTAGCTATGCGTCCAGCACAGGCAGACTATGATGCCTG. Antisense: CAAACCGGAGACTAGCGAGCAT.

### RNA pull-down assays

RNA pull-down assays and deletion mapping were conducted as described previously 9. Briefly, lnc-MRPL39-2:1 or antisense RNA were biotin-labeled using the Biotin RNA Labeling Mix (Roche) and transcribed* in vitr*o with T7 RNA polymerase (Roche, Indianapolis, USA). Next, the samples were treated using RNase-free DNase I (Roche) and purified with a RNeasy Mini Kit (Qiagen, Hilden, Germany). Biotinylated RNA was mixed with protein from CNE2 cell extracts and streptavidin agarose beads and incubated at room temperature for 1 h. Subsequently, the beads were boiled in sodium dodecyl sulfate (SDS) buffer and the pull-down proteins were analyzed by Western blotting. Finally, the SDS-polyacrylamide gel electrophoresis (PAGE) gel was silver-stained, and the important protein bands were extracted, cleaned, and analyzed by mass spectrometry (MS). Briefly, the gels were resuspended in digestion buffer with trypsin (Promega) and incubated for 20 h at 37°C. The peptides were then purified, dried, dissolved in 0.1% trifluoroacetic acid and validated using a Q Exactive mass spectrometer (Thermo). Trapping was performed at 10 μl/min for 4 min in 0.1% formic acid in water and the sample was loaded onto a reverse-phase column. The mass spectrometer was operated in data-dependent mode, automatically switching between MS and MS/MS acquisition. Finally, data analysis was performed using the Proteome Discoverer 1.4 software. The probes were as follows: TAATGTCCACTTGACCTCATCTATT; antisense: AATAGATGAGGTCAAGTGGACAT.

### Plasmid, siRNAs transfection, and quantitative real-time PCR Assays

The cells were transfected with siRNAs or plasmids with the help of the Lipofectamine 2000 reagent (Invitrogen) following the manufacturer's protocol. To check if the transfection was successful, the RNA expression was analyzed by qPCR. Briefly, we harvested total RNA from the cells or tissues using the TRIzol reagent (Invitrogen). Then, RNA was transcribed into complementary DNA (cDNA) with a cDNA Synthesis Kit (Promega). Expression analysis of RNA was carried out on an ABI 7500 PCR instrument using the SYBR Green PCR reagents (Life Technologies, Carlsbad, CA, USA) following the manufacturer's instructions. The researchers calculated the fold change by the 2^-ΔΔCt^ technique. *GAPDH* and *U6* were used as endogenous controls for quantifying the lncRNA and miRNA, respectively.

### FISH assay

The FISH Kit and lncRNA FISH Probe Mix (Ribo, Guangzhou, China) were used for FISH assays following the manufacturer's instructions (Guangzhou RiboBio Co., Ltd., Guangzhou, China). Briefly, cells seeded onto the slides in 24-well culture plates were fixed in 4% paraformaldehyde at room temperature for 30 mins. Cells were then cultured with a lncRNA probe and then counterstained with DAPI. Finally, cells were observed and images were captured on the laser scanning confocal microscope (Olympus, Tokyo, Japan). In this experiment, the researchers used U6 and 18S RNA as the controls for the nucleus and cytoplasmic subcellular localization, respectively.

### Cell proliferation assays

For the colony formation assay, NPC cells (exponential growth phase) were transfected with a vector containing the lncRNA or the control vector and seeded into the 6-well plates at the density of 1000 cells/well. The cells were allowed to grow for two weeks without disturbance. Finally, the colonies were fixed using methanol and then stained using crystal violet. The stained colonies were scanned by a high-resolution scanner, and the total number of colonies was quantified in each well. For the MTS assay, cells transfected with the lncRNA-expressing vector were introduced into the 96-well plates with 100 μL Dulbecco's modified eagle medium containing 10% FBS at the density of 5 × 10^3^ cells/well. MTS solution was added to every well, and after incubation at room temperature for 3 h, the absorbance of every well was detected at 490 nm, and the relative cell viability was calculated.

For the Click-iT® Plus EdU proliferation assay, cells were introduced into the 96-well plates and grown to 50-60% confluency. Then, cells were incubated with 10 µM EdU for 2 h. They were then reincubated with 100 µL 1× Apollo dyeing reaction solution at 4 ^o^C for 30 min. Subsequently, the cells were harvested and analyzed with the aid of flow cytometry.

### Wound closure and invasion assays

NPC cells in the exponential growth phase were seeded into 24-cell culture plates and incubated in RPMI 1640 medium supplemented with 5% (v/v) FBS until a confluent cell monolayer was formed. A wound was incised in the central area of the culture plate using a 10-μL pipette tip, and the detached cells were removed. The researchers then added the RPMI 1640 medium with a low serum to the plates and images of the wound closure were captured at 0, 24, and 48 h using an inverted microscope (Olympus, Japan). Finally, the wound width was analyzed with ImageJ software (National Institutes of Health, USA). Migration and invasion assays were performed using Transwell chambers (Falcon, BD, USA). Transfected cells in serum-free RPMI 1640 medium were seeded at 5 × 10^4^/mL into the upper chamber with/without Matrigel matrix precoating. RPMI 1640 medium supplemented with 10% FBS was added to the lower chamber. After fixing with methanol, the cells in the upper chamber were removed and the cells migrated to the lower chamber were stained using crystal violet. Photomicrographs of the stained cells were acquired with an inverted fluorescence microscope (Olympus, Japan).

### Western blotting analysis

Cells were suspended in the lysis buffer that contained a protease inhibitor cocktail (Sigma-Aldrich) and centrifuged at 15,000 × *g* for 12 min at 4 °C. Subsequently, the supernatant was harvested, and the total protein concentration was estimated using the BCA Protein Assay Kit (Pierce, Thermo Fischer Scientific). An aliquot (20 µg) of the total protein sample was separated by SDS-PAGE and then transferred to a polyvinylidene fluoride (PVDF) membrane (Merck Millipore, Schwalbach, Germany). The PVDF membrane was blocked with 5% non-fat dried milk in Tris-buffered saline with Tween-20 (TBST) and incubated in the presence of primary antibodies (1:1,000 dilution) overnight at 4°C. The membrane was then washed with TBST buffer and incubated with the corresponding HRP-conjugated secondary antibody for 1 h at room temperature. Finally, immunoreactive signals were visualized using the ECL reagent (Merck Millipore).

### Dual-luciferase reporter gene system

For lncRNA-MRPL39-2:1 and miR-329 luciferase assays, lncRNA-MRPL39-2:1 sequences containing wild-type (WT) or mutant (Mut) miRNA binding sites were synthesized and inserted into the pmirGLO luciferase vector (Genechem, Shanghai, China). The constructs were then co-transfected with miR-329 mimics into HEK293T cells using Lipofectamine3000 (Thermo Fisher). After 48 h, relative luciferase activity was measured using the dual-luciferase reporter gene system (Promega) according to the manufacturer's protocol. Relative luciferase activity was normalized to the Renilla luciferase internal control.

### Bioinformatics assay and statistical analysis

NPC‐related gene expression data set gene set enrichment (GSE) 53819 included 18 NPC tissue specimens and 18 non-cancerous tissue specimens. The array data for GSE12452 contains 41 samples, including 31 NPC and 10 normal nasopharyngeal tissues. Both datasets were retrieved from the Gene Expression Omnibus (GEO, https://www.ncbi.nlm.nih.gov/geo/). The expression of lncRNA-MRPL39-2:1 in tumor and non-tumor ovarian samples from the databases were analyzed using the R package.

### Gene ontology (GO) and KEGG enrichment analysis of RNA‑sequencing data

The functional roles of the differently expressed mRNAs and lncRNA target genes were investigated by GO enrichment analysis using the GOseq R package. DAVID software (https://david.ncifcrf.gov) was utilized to evaluate the statistical significance of the enrichment of differentially expressed mRNAs and lncRNA target genes in KEGG pathways (Kyoto Encyclopedia of Genes and Genomes). Two-sided or Bonferroni-corrected Fisher's exact tests were used to calculate the *P*-values of KEGG pathways or GO terms, respectively. A threshold of *P* < 0.05 and FDR ≤ 0.25 was applied to select significant items.

### LncRNA identification and potential miRNAs prediction

The PhyloCSF developed at the University of California Santa Cruz (UCSC) was utilized to analyze the coding potential of lnc-MRPL39-2:1 transcripts. Three other online software packages, LNCipedia, RegRNA 2.0 and the Coding Potential Assessment Tool (CPAT), were also used for potential noncoding analysis. The online LNCipedia (https://lncipedia.org/), DIANA tool (http://diana.imis.athena-innovation.gr) and TargetScan (http://www.targetscan.org) were used to screen potential miRNAs that may bind with the lnc-MRPL39-2:1 transcript.

### IHC Analysis

The tissue samples from humans or mice were fixed in 4% (v/v) paraformaldehyde solution for 48 h. Then, the 4 μm-thick paraffin-embedded tissue sections were deparaffinized using xylene and rehydrated using a graded ethanol series. The sections were incubated in the blocking solution for 10 mins and then incubated again in the presence of corresponding primary antibodies at 4 °C overnight. Next, these sections were incubated using secondary antibodies for 1 h, counterstained with hematoxylin (Sigma-Aldrich), and imaged by a light microscope (Olympus, Japan). The percentage of the positive cells and staining intensity were analyzed by two independent observers.

### *In vivo* mice studies

The animal experiments were approved by the Medical Ethics committee at Guangzhou Medical University (Guangzhou, China). To validate the tumorigenesis and metastasis of lnc-MRPL39-2:1* in vivo*, the researchers purchased six-week-old female BALB/c(nu/nu) nude mice from the Guangzhou Medical University. The researchers investigated the lnc-MRPL39-2:1-induced tumor growth *in vivo* by means of subcutaneously injecting lentiviral-infected NPC cells (5×10^6^/100 µL) into the right upper flank of the mouse, and measured the tumor volume every 3 days for 30 days. To validate the metastatic ability promoted by the lnc-MRPL39-2:1, cells infected with lentivirus carrying only vector or lnc-MRPL39-2:1 (2×10^6^ cells in 0.2 mL phosphate-buffered saline) were injected into the tail veins of the mice, and the tumors formed in the lung were monitored using a bioluminescence imaging system. Finally, six weeks after injecting the cells, the mice were euthanized, and the number of tumors in the lung was observed and stained with H&E.

### Statistical analysis

Statistical analyses were carried out using the SPSS ver. 23.0 software (IBM Corp., Armonk, NY, USA). All data acquired from the *in vitro* experiments were expressed as the mean value ± SD based on three or more independent experiments. The researchers employed the Kaplan-Meier technique and Cox proportional hazard regression model for the purpose of analyzing the OS. They further used the chi-square test and Fisher's exact test for analyzing the relationship between the lncRNAs and other clinicopathological parameters, including age, clinical stage, and tumor size. A *P*-value<0.05 was considered to be statistically significant.

## Supplementary Material

Supplementary figures and tables.

## Acknowledgments and Funding

This work was supported by the National Natural Science Foundation of China (No. 81872195, No. 82102974, No. 81803170), the Guangzhou Key Medical Discipline Construction Project Fund, and the Key Clinical Technology of Guangzhou (2019D17).

## References

[1] Chua MLK, Wee JTS, Hui EP, Chan ATC (2016). Nasopharyngeal carcinoma. Lancet (London, England).

[2] Chen YP, Chan ATC, Le QT, Blanchard P, Sun Y, Ma J (2019). Nasopharyngeal carcinoma. Lancet (London, England).

[3] Lee AW, Ma BB, Ng WT, Chan AT (2015). Management of Nasopharyngeal Carcinoma: Current Practice and Future Perspective. Journal of clinical oncology: official journal of the American Society of Clinical Oncology.

[4] Hong X, Liu N, Liang Y, He Q, Yang X, Lei Y (2020). Circular RNA CRIM1 functions as a ceRNA to promote nasopharyngeal carcinoma metastasis and docetaxel chemoresistance through upregulating FOXQ1. Molecular cancer.

[5] Zheng ZQ, Li ZX, Zhou GQ, Lin L, Zhang LL, Lv JW (2019). Long Noncoding RNA FAM225A Promotes Nasopharyngeal Carcinoma Tumorigenesis and Metastasis by Acting as ceRNA to Sponge miR-590-3p/miR-1275 and Upregulate ITGB3. Cancer Res.

[6] Kopp F, Mendell JT (2018). Functional Classification and Experimental Dissection of Long Noncoding RNAs. Cell.

[7] Liu SJ, Dang HX, Lim DA, Feng FY, Maher CA (2021). Long noncoding RNAs in cancer metastasis. Nature reviews Cancer.

[8] Statello L, Guo CJ, Chen LL Gene regulation by long non-coding RNAs and its biological functions. 2021; 22: 96-118.

[9] Wang B, Zheng J, Li R, Tian Y, Lin J, Liang Y (2019). Long noncoding RNA LINC02582 acts downstream of miR-200c to promote radioresistance through CHK1 in breast cancer cells. Cell death & disease.

[10] Zheng ZQ, Li ZX, Zhou GQ, Lin L, Zhang LL, Lv JW Long Noncoding RNA FAM225A Promotes Nasopharyngeal Carcinoma Tumorigenesis and Metastasis by Acting as ceRNA to Sponge miR-590-3p/miR-1275 and Upregulate ITGB3. 2019; 79: 4612-26.

[11] Wang Y, Chen W, Lian J, Zhang H, Yu B The lncRNA PVT1 regulates nasopharyngeal carcinoma cell proliferation via activating the KAT2A acetyltransferase and stabilizing HIF-1α. 2020; 27: 695-710.

[12] Ale-Agha N, Galban S, Sobieroy C, Abdelmohsen K, Gorospe M, Sies H (2009). HuR regulates gap junctional intercellular communication by controlling beta-catenin levels and adherens junction integrity. Hepatology (Baltimore, Md).

[13] Cao C, Sun J, Zhang D, Guo X, Xie L, Li X (2015). The long intergenic noncoding RNA UFC1, a target of MicroRNA 34a, interacts with the mRNA stabilizing protein HuR to increase levels of beta-catenin in HCC cells. Gastroenterology.

[14] Li N, Yang G, Luo L, Ling L, Wang X, Shi L (2020). lncRNA THAP9-AS1 Promotes Pancreatic Ductal Adenocarcinoma Growth and Leads to a Poor Clinical Outcome via Sponging miR-484 and Interacting with YAP. Clinical cancer research: an official journal of the American Association for Cancer Research.

[15] Luo L, Zhang J, Tang H, Zhai D, Huang D, Ling L (2020). LncRNA SNORD3A specifically sensitizes breast cancer cells to 5-FU by sponging miR-185-5p to enhance UMPS expression. Cell death & disease.

[16] Peng WX, Koirala P, Mo YY (2017). LncRNA-mediated regulation of cell signaling in cancer. Oncogene.

[17] Zhou L, Liu R, Liang X, Zhang S, Bi W, Yang M (2020). lncRNA RP11-624L4.1 Is Associated with Unfavorable Prognosis and Promotes Proliferation via the CDK4/6-Cyclin D1-Rb-E2F1 Pathway in NPC. Molecular therapy Nucleic acids.

[18] Zheng ZQ, Li ZX, Guan JL, Liu X, Li JY, Chen Y Long Noncoding RNA TINCR-Mediated Regulation of Acetyl-CoA Metabolism Promotes Nasopharyngeal Carcinoma Progression and Chemoresistance. 2020; 80: 5174-88.

[19] Dong R, Chen P, Polireddy K, Wu X, Wang T, Ramesh R (2020). An RNA-Binding Protein, Hu-antigen R, in Pancreatic Cancer Epithelial to Mesenchymal Transition, Metastasis, and Cancer Stem Cells. Molecular cancer therapeutics.

[20] Cai J, Wang H, Jiao X, Huang R, Qin Q, Zhang J (2019). The RNA-Binding Protein HuR Confers Oxaliplatin Resistance of Colorectal Cancer By Upregulating CDC6. Molecular cancer therapeutics.

[21] Hu YP, Jin YP, Wu XS, Yang Y, Li YS, Li HF (2019). LncRNA-HGBC stabilized by HuR promotes gallbladder cancer progression by regulating miR-502-3p/SET/AKT axis. Molecular cancer.

