# Supplementary material for: Upregulated Long Non-coding RNA Lnc-MRPL39-2:1 Induces the Growth and Invasion of Nasopharyngeal Carcinoma by Binding to HuR and Stabilizing β-Catenin mRNA
Source: Int J Biol Sci. 2023 Apr 25;19(8):2349–65. doi: 10.7150/ijbs.79115 (PMC10197890; doi:10.7150/ijbs.79115)

Supplementary Data:

Table S1: Clinical characteristics of nasopharyngeal carcinoma patients with high or low expression of lnc-MRPL39-2:1.

Table S2: The relationship between lnc-MRPL39-2:1 expression and survival analyzed by univariate and multivariable Cox regression analysis.

Table S3: miRNAs targeted to lnc-MRPL39-2:1 analyzed using the human lncRNA database LNCipedia (https://lncipedia.org/).

Fig. S1: Analysis of the differentially expressed lncRNAs and mRNAs between the NPC tumor tissues and para-tumor tissues. (**A** and **B**) Scatter plot of differentially expressed lncRNAs (**A**) and mRNAs (**B**) Between tumor and para-tumor tissues. Green lines represent fold change. (**C** and **D**) Volcano plots show differentially expressed lncRNAs (**C**) and Gene (**B**) in NPC and para-tumor tissues. Red plots represent significantly upregulated lncRNAs and mRNAs.

Fig. S2: Differentially expressed mRNAs were functionally classified via GO functional enrichment analysis and pathway annotation. The downregulated mRNAs were classified by biological processes (**A**) Cellular components (**B**)Molecular function (**C**) GO terms. The biological processes (**D**) Cellular components (**E**), and Molecular function (**F**) of the upregulated mRNAs in the NPC compared to para-tumor tissue groups. Pathway analyses of upregulated (**G**) and Downregulated mRNAs (**H**).

Fig. S3: Differentially expressed lncRNAs classified according to their distribution. (**A**) Dysregulated lncRNAs classified into intergenic, antisense overlapping, sense-overlapping, and bidirectional. (**B**) Differentially expressed lncRNAs distributed in various chromosomes. (**C**) Length distribution of the dysregulated lncRNAs.

Fig. S4: Differentially expressed lncRNAs in tissues and cells. (**A, B**) Expression of four upregulated (**A**) and three downregulated (**B**) lncRNAs measured by qPCR in five tumor and paired non-tumor tissues and NP69. *U6* was used as the loading control. The expression of lnc-MRPL39-2:1 was analyzed by RNA-FISH (**C**) and ISH assay (**D**).

Fig. S5: Full-length lnc-MRPL39-2:1 gene cloning. Gel electrophoresis (left panel) and sequencing (right panel) of nested PCR products from 3′ RACE **(A)** and 5′ RACE **(B)**, respectively. Arrows indicate the purpose band. **(C)** Full-length human lnc-MRPL39-2:1 transcript.

Fig. S6: Lnc-MRPL39-2:1 promotes NPC cell growth and migration. (**A** and **B**) Lnc-MRPL39-2:1 suppression remarkably inhibits HONE1 cell metastasis. (**A**) Trans-well migration and

invasion assays were performed to determine the impact of lnc-MRPL39-2:1 on the migration and invasion of HONE1 cells. (**B**) Wound-healing assays in HONE1 cells transfected with shRNA-lnc-MRPL39-2:1 or empty vector, related to **Fig. 3D**. (**C**) Lnc-MRPL39-2:1 suppression significantly inhibits NPC cell colony formation, related to **Fig. 3H**. (**D**) Expression of lnc-MRPL39-2:1 as validated by qPCR. Effects of lnc-MRPL39-2:1 overexpression on proliferation (**E**) and colony-forming ability (**F**) were tested in CNE2 and HONE1 cells, related to **Fig. 3O** and **Fig. 3P**, respectively. (**G**) Apoptosis was analyzed using flow cytometry (statistics shown in **Fig. 3Q**) ($^{*}P < 0.05$, $^{**}P < 0.01$).

Fig. S7: HuR is responsible for lnc-MRPL39-2:1-mediated cell growth and migration. (**A**) HuR in cancer and para-cancer tissues was tested using IHC. (**B**) OS and PFS in patients with low or high HuR expression were analyzed. (**C**) The proliferation ability of NPC cells was verified using EDU assay, related to **Fig. 4G**. (**D**) The number of colonies of NPC cells was measured by the colony-formation assay, related to **Fig. 4H**. (**E**) Apoptotic NPC cells with lnc-MRPL39-2:1 or/and siHuR were tested using flow cytometry, related to **Fig. 4I**.

Fig. S8: β-Catenin pathway is involved with HuR in NPC tissue. (**A**) OS and PFS in the patients with a low or high expression of β-Catenin were analyzed. (**B**) Correlation between HuR and β-Catenin expression was measured in 30 NPC samples by qPCR. (**C**) The relationship between HuR and β-Catenin was validated using IHC in primary NPC tissue.

Fig. S9: The tumor samples were verified by the hematoxylin and eosin staining. The expression of Ki-67 and lnc-MRPL39-2:1 in the cancer tissue was tested by IHC and ISH, respectively.

Table S1

**Characteristics**	**No. of patients**	**Expression of *Lnc-MRPL39-2***		***P* value**
	**n= 122**	**Low, *n* (%)**	**High, *n* (%)**	
**Age**				
≤40	46	11	35	1
>40	76	19	57	
**Sex**				
Male	87	23	64	0.497
Female	35	7	28	
**T Stage**				
T1-T2	43	17	26	**0.008**
T3-T4	79	13	64	
**N Stage**				
N0-N1	57	22	35	**0.01**
N2-N3	65	8	57	
**M Stage**				
M0	103	26	77	1
M1	19	4	15	
**TNM Stage**				
I-II	43	23	20	**< 0.01**
III-IV	79	7	72	
**Local Failure**				
Yes	27	8	19	0.613
No	95	22	73	
**Regional Failure**				
Yes	23	5	18	0.793
No	99	26	73	

A. All patients were restaged according to the 8th edition of the AJCC Cancer Staging Manual. Bold values indicate $P<0.05$. P value is determined by χ^2 and Fisher's exact tests.

Table S2

Parameters	Univariate analysis			Multivariate analysis		
	**HR**	**95% CI**	***P***	**HR**	**95% CI**	***P***
			**Overall survival**			
Gender						
Male versus Female	1.32	0.95-2.45	0.41			
Age (years)						
≥40 *vs.* <40	1.23	0.85-1.62	0.36			
Histologic subtype						
DNKC *vs.* UDC	0.95	0.83-1.67	0.73			
TNM Stage						
III-IV *vs.* I-II	2.36	2.12-4.67	0.006	2.48	2.20-4.89	< 0.01
Lnc-MRPL39-2 expression						
High *vs.* Low	3.27	1.89-6.29	< 0.01	3.38	1.97-6.86	< 0.01
			**Progression Free Survival**			
Gender						
Male versus Female	2.32	0.76-3.10	0.34			
Age (years)						
≥40 *vs.* <40	1.89	0.67-1.91	0.46			
Histologic subtype						
DNKC *vs.* UDC	1.06	0.76-1.93	0.67			
TNM stage						
III-IV *vs.* I-II	1.96	1.45-4.32	0.003	2.23	2.05-5.21	0.01
Lnc-MRPL39-2 expression						
High *vs.* Low	4.01	2.24-8.36	< 0.01	4.31	2.68-7.93	< 0.01

Table S3

microRNA	MirTarget2 Score
has-miR-3613-3p	90.1
has-miR-3688-3p	89.75
has-miR-561-3p	89.57
has-miR-4775	88.72
has-miR-1283	86.78
has-miR-524-5p	86.38
has-miR-590-3p	83.21
has-miR-520d-5p	83.16
has-miR-144-5p	81.08
has-miR-4776-3p	77.88
has-miR-4668-3p	76.24
has-miR-548at-5p	76.07
has-miR-4769-3p	75.54
has-miR-934	74.13
has-miR-329	73.37
has-miR-362-3p	72.02
has-miR-16-1-3p	67.57
has-miR-551b-5p	67.4
has-miR-569	66.14
has-miR-3125	65.52
has-miR-875-5p	64.63
has-miR-187-5p	63.03
has-miR-4777-3p	60.64
has-miR-2053	60.36
has-miR-4763-3p	59.95
has-miR-501-5p	59.23
has-miR-4787-59	58.73
has-miR-3169	58.51
has-miR-518a-5p	57.17
has-miR-527	57.17
has-miR-4802-3p	56.77
has-miR-376c	56.25
has-miR-32-3p	55.7
has-miR-4690-5p	55.46
has-miR-1207-5p	54.15
has-miR-877-5p	53.84
has-miR-567	53.34
has-miR-592	52.37
has-miR-3916	52.35

Fig. S1

A

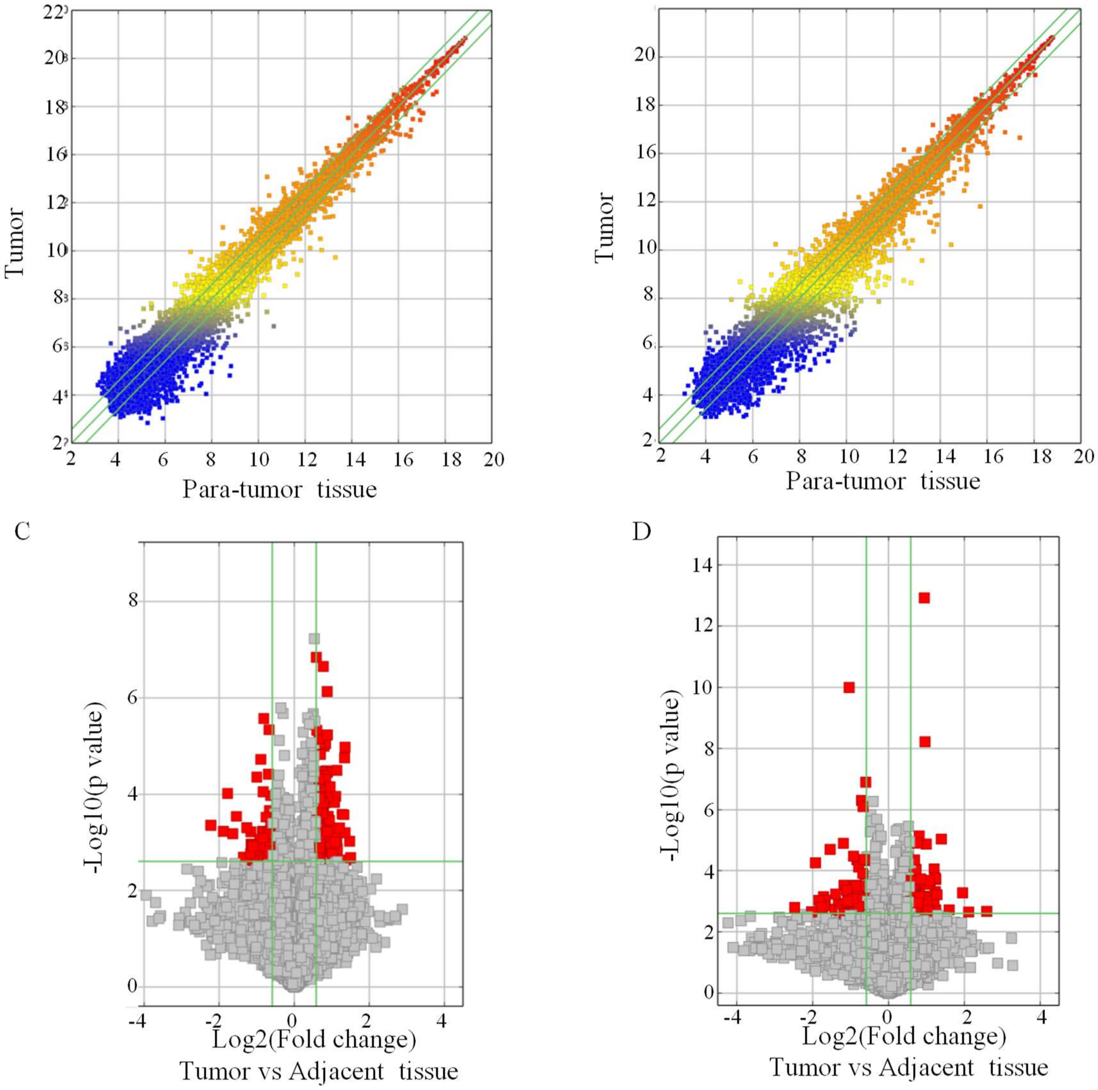
Tumor
Para-tumor tissue
22
20
18
16
12
10
8
6
4
2
2 4 6 8 10 12 14 16 18 20
Tumor
Para-tumor tissue
20
18
16
12
10
8
6
4
2
2 4 6 8 10 12 14 16 18 20
C
-Log10(p value)
Log2(Fold change)
Tumor vs Adjacent tissue
8
6
4
2
0
-4 -2 0 2 4
D
-Log10(p value)
Log2(Fold change)
Tumor vs Adjacent tissue
14
12
10
8
6
4
2
0
-4 -2 0 2 4

Fig. S2

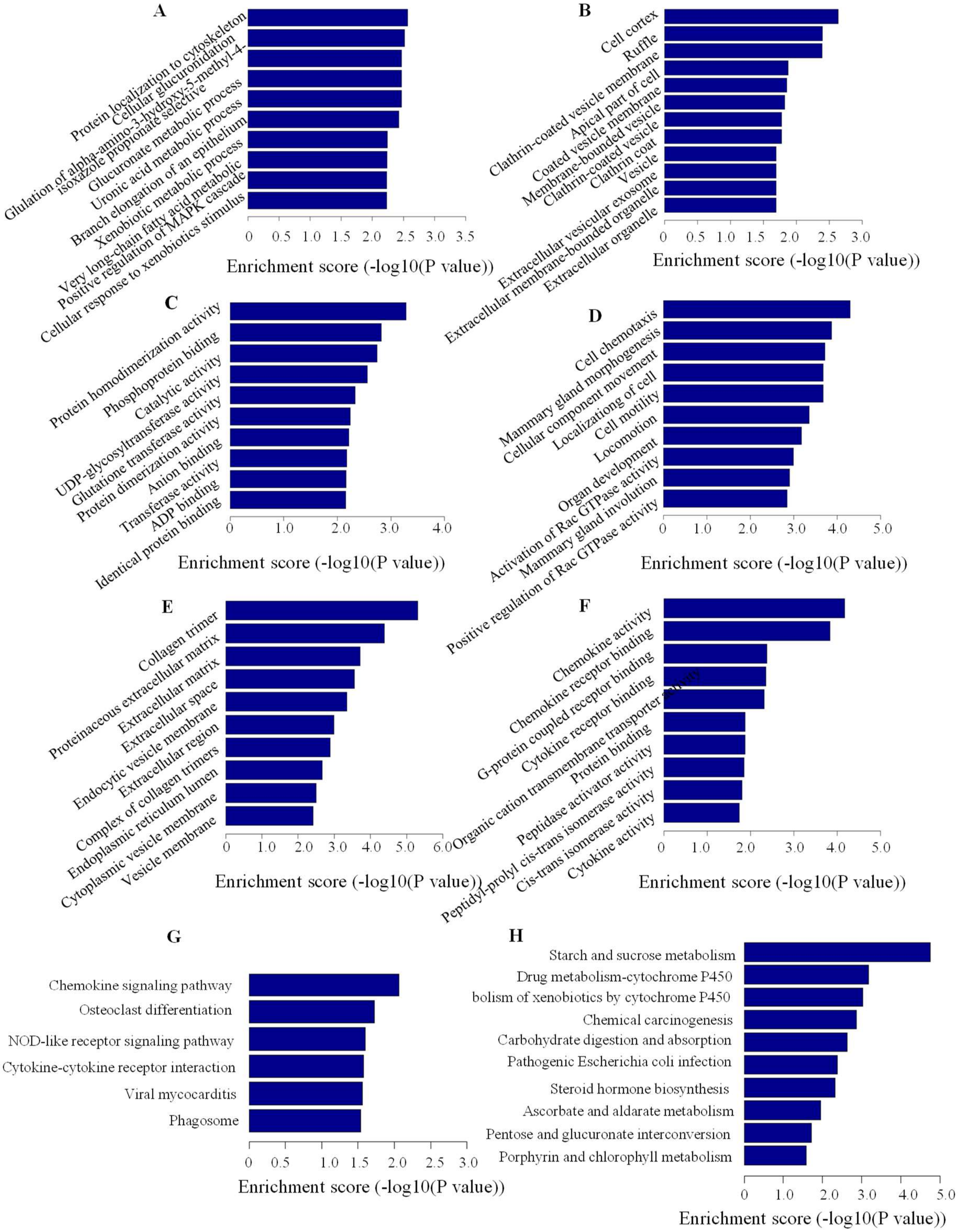
A
Protein localization to cytoskeleton
Cellular glucuronidation
Glulation of alpha-amino-3-hydroxy-5-methyl-4-isoxazole propionate selective
Glucuronate metabolic process
Uronic acid metabolic process
Branch elongation of an epithelium
Xenobiotic metabolic process
Very long-chain fatty acid metabolic
Positive regulation of MAPK cascade
Cellular response to xenobiotics stimulus
0 0.5 1.0 1.5 2.0 2.5 3.0 3.5
Enrichment score (-log10(P value))
B
Cell cortex
Ruffle
Clathrin-coated vesicle membrane
Apical part of cell
Coated vesicle membrane
Membrane-bounded vesicle
Clathrin-coated vesicle
Clathrin coat
Vesicle
Extracellular vesicular exosome
Extracellular membrane-bounded organelle
Extracellular organelle
0 0.5 1.0 1.5 2.0 2.5 3.0
Enrichment score (-log10(P value))
C
Protein homodimerization activity
Phosphoprotein biding
Catalytic activity
UDP-glycosyltransferase activity
Glutatione transferase activity
Protein dimerization activity
Anion binding
Transferase activity
ADP binding
Identical protein binding
0 1.0 2.0 3.0 4.0
Enrichment score (-log10(P value))
D
Cell chemotaxis
Mammary gland morphogenesis
Cellular component movement
Localizationg of cell
Cell motility
Locomotion
Organ development
Activation of Rac GTPase activity
Mammary gland involution
Positive regulation of Rac GTPase activity
0 1.0 2.0 3.0 4.0 5.0
Enrichment score (-log10(P value))
E
Collagen trimer
Proteinaceous extracellular matrix
Extracellular matrix
Extracellular space
Endocytic vesicle membrane
Extracellular region
Complex of collagen trimers
Endoplasmic reticulum lumen
Cytoplasmic vesicle membrane
Vesicle membrane
0 1.0 2.0 3.0 4.0 5.0 6.0
Enrichment score (-log10(P value))
F
Chemokine activity
Chemokine receptor binding
G-protein coupled receptor binding
Cytokine receptor binding
Organic cation transmembrane transporter activity
Protein binding
Peptidase activator activity
Peptidyl-prolyl cis-trans isomerase activity
Cis-trans isomerase activity
Cytokine activity
0 1.0 2.0 3.0 4.0 5.0
Enrichment score (-log10(P value))
G
Chemokine signaling pathway
Osteoclast differentiation
NOD-like receptor signaling pathway
Cytokine-cytokine receptor interaction
Viral mycocarditis
Phagosome
0 0.5 1.0 1.5 2.0 2.5 3.0
Enrichment score (-log10(P value))
H
Starch and sucrose metabolism
Drug metabolism-cytochrome P450
bolism of xenobiotics by cytochrome P450
Chemical carcinogenesis
Carbohydrate digestion and absorption
Pathogenic Escherichia coli infection
Steroid hormone biosynthesis
Ascorbate and aldarate metabolism
Pentose and glucuronate interconversion
Porphyrin and chlorophyll metabolism
0 1.0 2.0 3.0 4.0 5.0
Enrichment score (-log10(P value))

Fig. S3

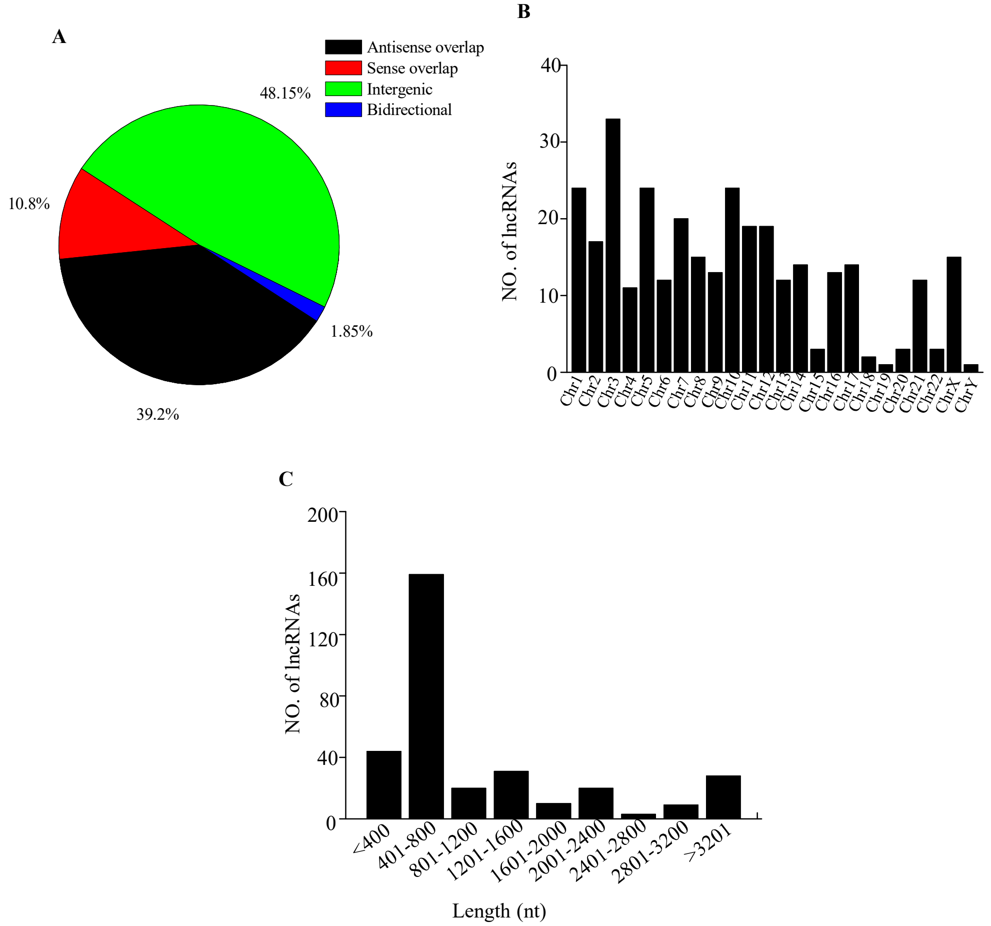
A
Antisense overlap
Sense overlap
Intergenic
Bidirectional
48.15%
10.8%
1.85%
39.2%
B
NO. of lncRNAs
0
10
20
30
40
Chr1
Chr2
Chr3
Chr4
Chr5
Chr6
Chr7
Chr8
Chr9
Chr10
Chr11
Chr12
Chr13
Chr14
Chr15
Chr16
Chr17
Chr18
Chr19
Chr20
Chr21
Chr22
ChrX
ChrY
C
NO. of lncRNAs
0
40
80
120
160
200
<400
401-800
801-1200
1201-1600
1601-2000
2001-2400
2401-2800
2801-3200
>3201
Length (nt)

Fig. S4

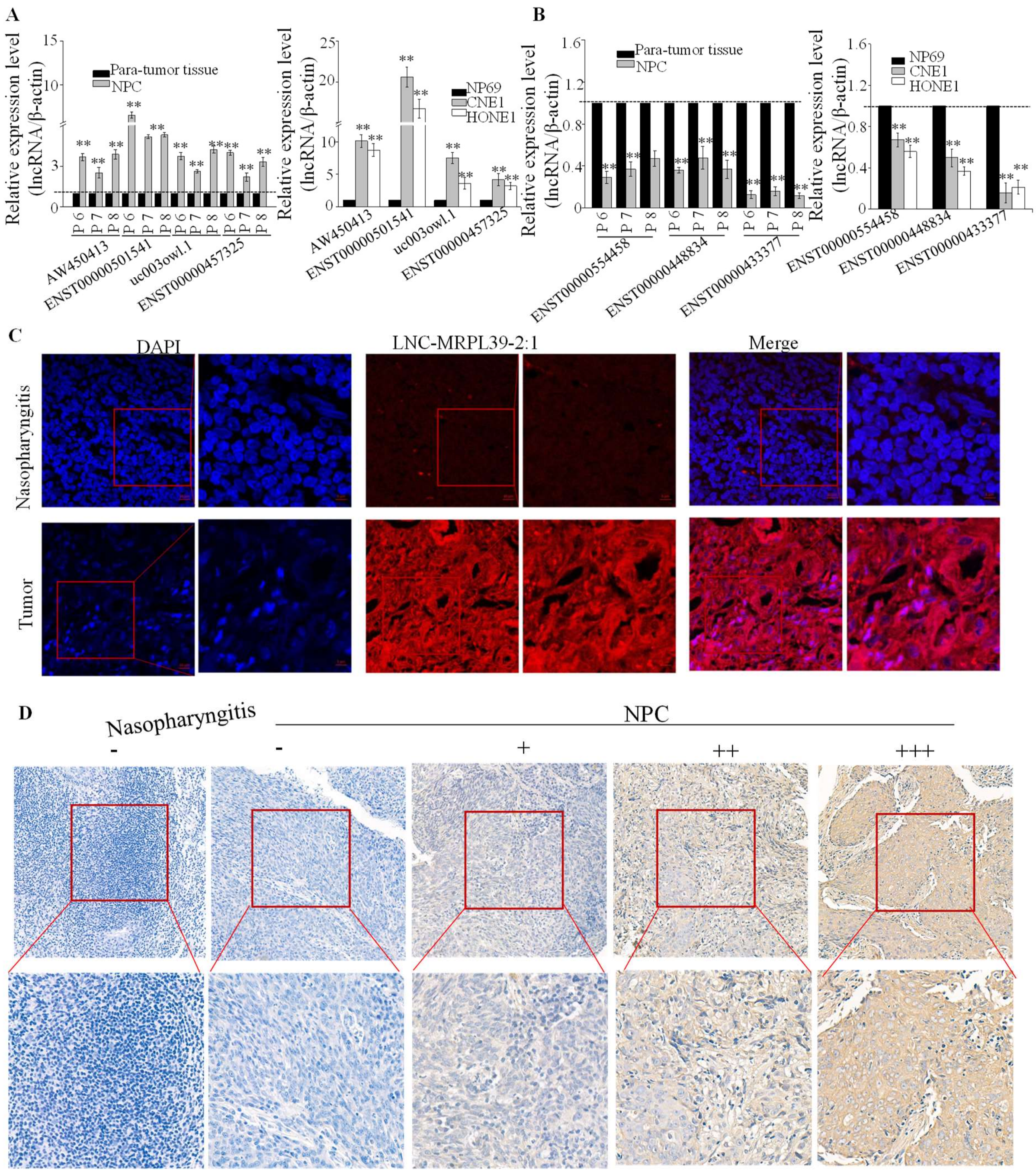
A
Relative expression level (lncRNA/β-actin)
Para-tumor tissue
NPC
P 6
P 7
P 8
AW450413
ENST00000501541
uc003owl.1
ENST00000457325
NP69
CNE1
HONE1
B
ENST00000554458
ENST00000448834
ENST00000433377
C
DAPI
LNC-MRPL39-2:1
Merge
Nasopharyngitis
Tumor
D
Nasopharyngitis
NPC
-
+
++
+++

Fig. S5

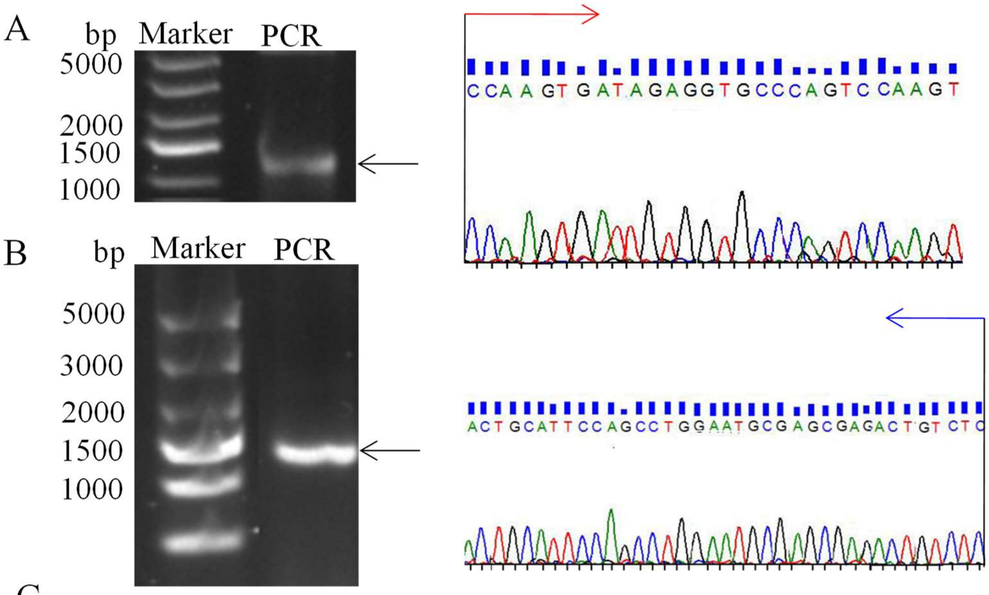

C

5'CCAAGTGATAGAGGTGCCCAGTCCAAGTGAAGGGCAAGAGTCTACATACCACTTTCCTCTATTCAAGCTATTTATGCCTACTAGGTTTAGAATACTGATTAT
GAAAGTGGACCCCCGAAGAGGGTCATGGCTGAGATTCAAGGAAGTCTCTAGCCTGCTAACATCGAATGCAAATTGGTGTGTGAGGATACACATTAACAAA
AATTTTCTGGGAAGAGCATATAAAATTTCCATTAGATTCTCAAGGAGGTAGGAATAAAGGATTTTAGTAACTATTACTCTAAAATGAAAGAATTCCTTTAAAT
TGAATGGCAGATCTTAGTCATGACCTGAAAAGCTGATAAAAAAAAACTTTTCAAAAGTTAAAACCTAGGGCTTTTTGGGGTAGATTTTTTGATTTATAGAG
GACTGGTAAGATGAGGTATTTAAAGATGAGGGTTACAAATTTTCCATGTTGGCATATGGAAAGTAAAGAATGGCAAAATTTGTAGCACATTCTAATTAGTTC
AAATATCTATGTTACCAAAATTGAAATGATATCAAATTGCACAGCAATGTCCTGATGGCCTTTCCTCTAACTTTCTGGGTTTGTAAGGGTAGGGTTGTGCCTAT
CCTGCTCACCTTTGTCTCTCCAGTGTCTAGCATAGCGTCCAGCACAGGCAGACTATGATGCCTGTTTTTTTTTTGTTGTTGTTTTTTTTTTTTTTTTTTTGGTA
AAGACCAGTGAATTACACTTTTTACACATGACTAGGTTAAAACAAAAGTAGACATTTGTGCAAACACCACTGTTCTCCCTTGAAGTGGTTGTAGGTCTCAG
ACAGTGCTAATTTTTCTCTTTCTTAATGTGTGTGGTTAGGAAGCTGGGTCCTGTGGCCAGAGTGAGCTGACCCACTATGTGAGGAAACTTACTTATCACAGC
CTAATAAGGAGTAGTCCTTGCAGCAGAAGTAATTACTAAATGTGCTTTGGTCATTTGACAGAAATCAGGGAGGCAGCAAGGAGTCATGGAAATGAGCCCA
AACTGAGAGTCAAAACCCTGTGGTCTGCCGACACCCCCATCCCACATCCGTACCTAAAAGCAGGCTGTGCACTAGGGACCTAGTGACCTTACTAGAAAAA
ACTCAAATTCTCTGAGCCACAAGTCCTCATGGGCAAAATGTAGATACCACCACCTAACCCTGCCAATTTCCTATCATTGTGACTATCAAATTAAACCACAGGC
AGGAAGTTGCCTTGAAAACTTTTTATAGTGTATATTACTGTTCACATAGATAAGCAATTAACTTTACATATACCCGTTTTTAAAAGATCAGTCCTGTGATTAAA
AGTCTGGCTGCCCTAATTCACTTCGATTATACATTAGGTTAAAGCCATATAAAAGAGGCACTACGTCTTCGGAGAGATGAATGGATATTACAAGCAGTAATTT
TGGCTTTGGAATATACACATAATGTCCACTTGACCTCATCTATTTGACACAAAATGTAAACTAAATTATGAGCATCATTAGATACCTTGGCCTTTTCAAATCACA
CAGGGTCCTAGATCTGCTGTGTGATGCTGGAAAAGTAACTTCACTTCTGTGGGCCTCAGCTCCTTAATTTTTAACATACTTTGGATTCTTATATCTTTGTCAGC
TGTCAACTTCAGTGTTTTCAGGTTAAATTCTATCCATAGTCATCCCAATATACCTGCTTTAGATGATACAAACTTCAAAAGATCCGGCTCTCCCTCGTAAAACG
TGGAGGACAGACATCAAGGGGGTTTTGTGAGTAAAGAAAGGCAACCGCTCGGCAAAAACGCACCCTGGCACAACAGGAGCGAATAAATACAGACGCTG
ATTGAGCGTTTGCTCCATCTTCACTTCTGTTAAATGAAGACATTGATATCTAAAATGCTATGAGTCTAACTTTGTAAAATTAAAATAGATTTGTAGTTATTTTTC
AAAATGAAATCGAAAAGATACAAGTTTTGAAGGCAGTCTCTTTTTCCACCCTGCCCCTCTAGTGTGTTTTACACACTTCTCTGGCCACTCCAACAGGGAAG
CTGGTCCAGGGCCATTATACAGGTACGTCCCCAACCATCCAAGGAAACTTTGCAAAGAAGCTCAAGTATTAGGTTTCGAACACCCCTCCTTCCCCCTGCCTC
TGCCCGCGGGAATCCATCCAGCACTTTGGGAGGCTGAGGTGACTGGATCATGAGGTCAGGAGATTGAGACCATCCTGGCTAGAATGGTGAAACCCTGAA
TCTACTAAAAATACAAAAAAGAAAAAAAAATTAGCCGGGCATGATGGCGGGCACCTGTAGTCCCAGCTACTCTGGAGGCTGAGGCAGGAGAATGGCATG
AATGCGGGAGGCGGAGTTTGCGGTGAGCCAAGATCGCGCCACTGCATTCCAGCCTGGAATGCGAGCGAGACTGTCTC3'

Fig. S6

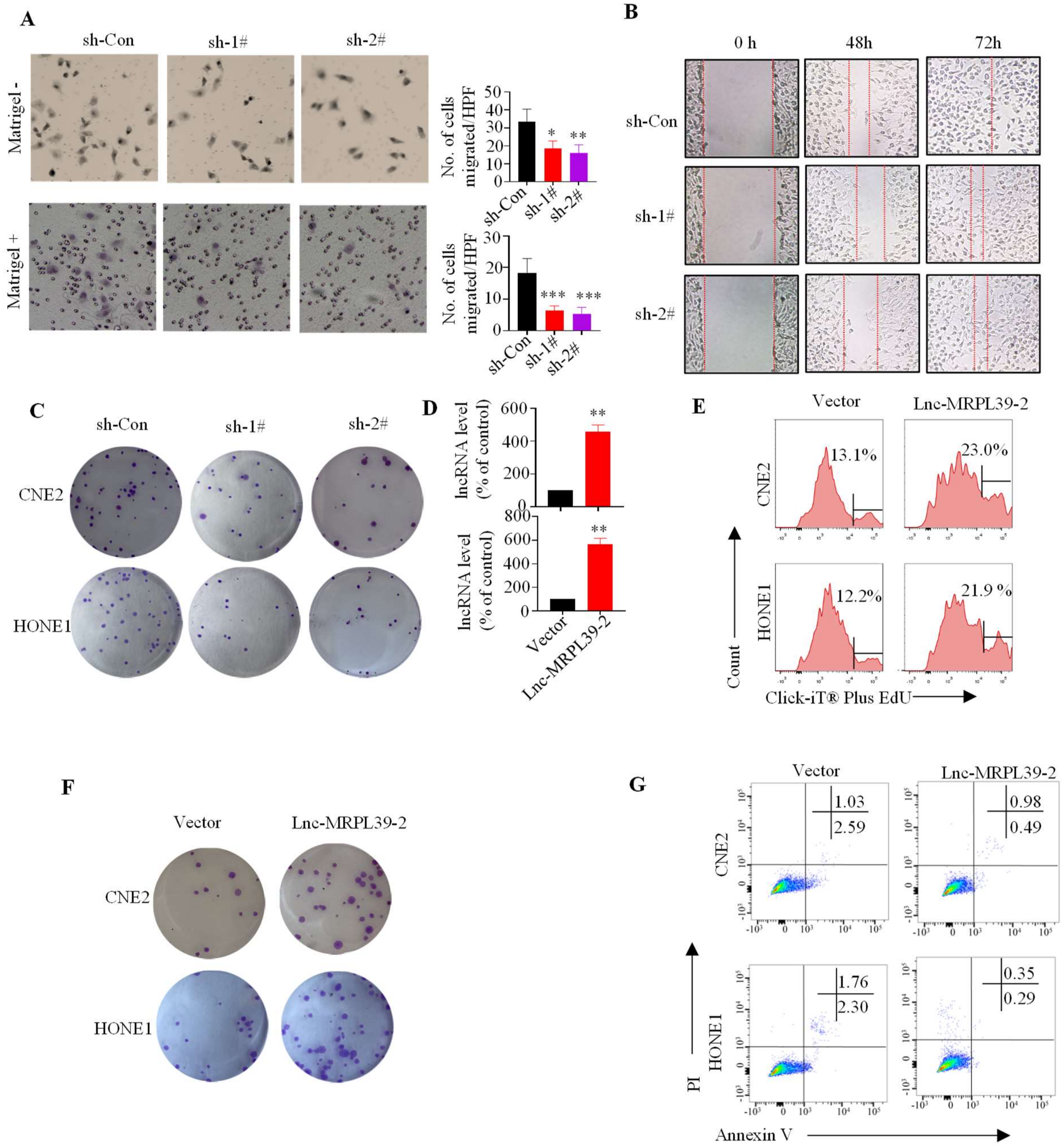

A
sh-Con
sh-1#
sh-2#
Matrigel -
Matrigel +
No. of cells migrated/HPF
B
0 h
48h
72h
C
CNE2
HONE1
D
lncRNA level (% of control)
Vector
Lnc-MRPL39-2
E
Vector
Lnc-MRPL39-2
13.1%
23.0%
12.2%
21.9 %
Count
Click-iT® Plus EdU
F
G
1.03
2.59
0.98
0.49
1.76
2.30
0.35
0.29
PI
Annexin V

Fig. S7

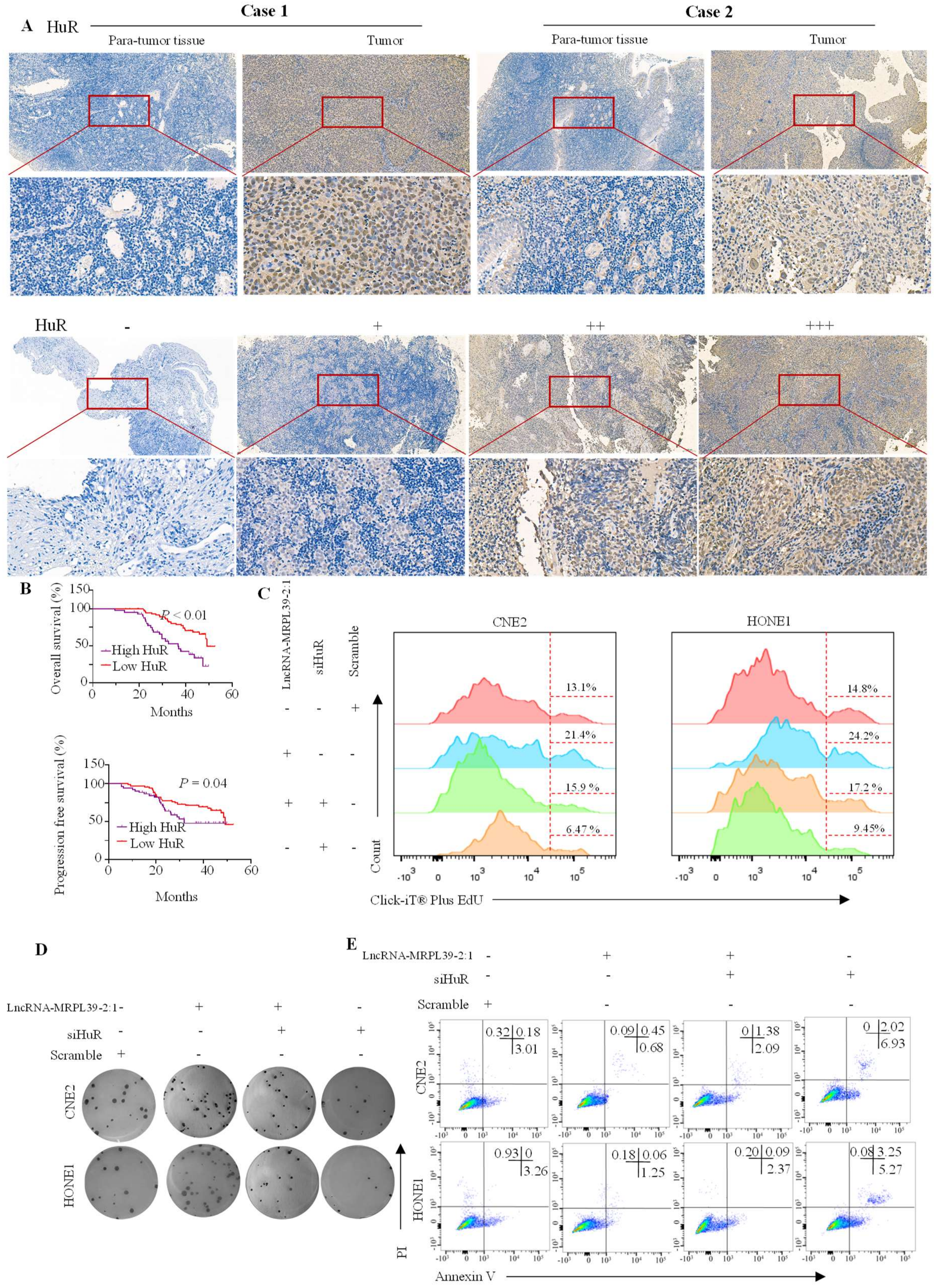
A HuR
Case 1
Case 2
Para-tumor tissue
Tumor
Para-tumor tissue
Tumor
HuR - + ++ +++
B
Overall survival (%)
P < 0.01
High HuR
Low HuR
Months
Progression free survival (%)
P = 0.04
High HuR
Low HuR
Months
C
LncRNA-MRPL39-2:1
siHuR
Scramble
CNE2
HONE1
13.1%
21.4%
15.9 %
6.47 %
14.8%
24.2%
17.2 %
9.45%
Count
Click-iT® Plus EdU
D
LncRNA-MRPL39-2:1
siHuR
Scramble
CNE2
HONE1
E
LncRNA-MRPL39-2:1
siHuR
Scramble
CNE2
HONE1
0.32 0.18 3.01
0.09 0.45 0.68
0 1.38 2.09
0 2.02 6.93
0.93 0 3.26
0.18 0.06 1.25
0.20 0.09 2.37
0.08 3.25 5.27
PI
Annexin V

Fig. S8

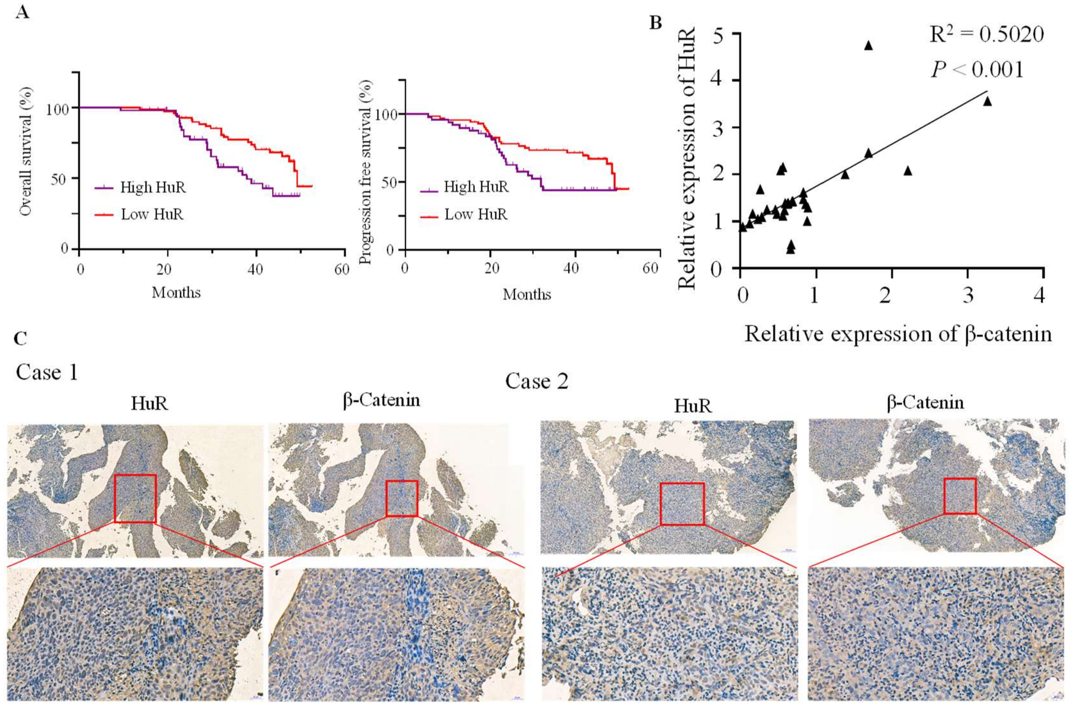
A
Overall survival (%)
Months
High HuR
Low HuR
Progression free survival (%)
B
Relative expression of HuR
Relative expression of β-catenin
R2 = 0.5020
P < 0.001
C
Case 1
Case 2
HuR
β-Catenin

Fig. S9

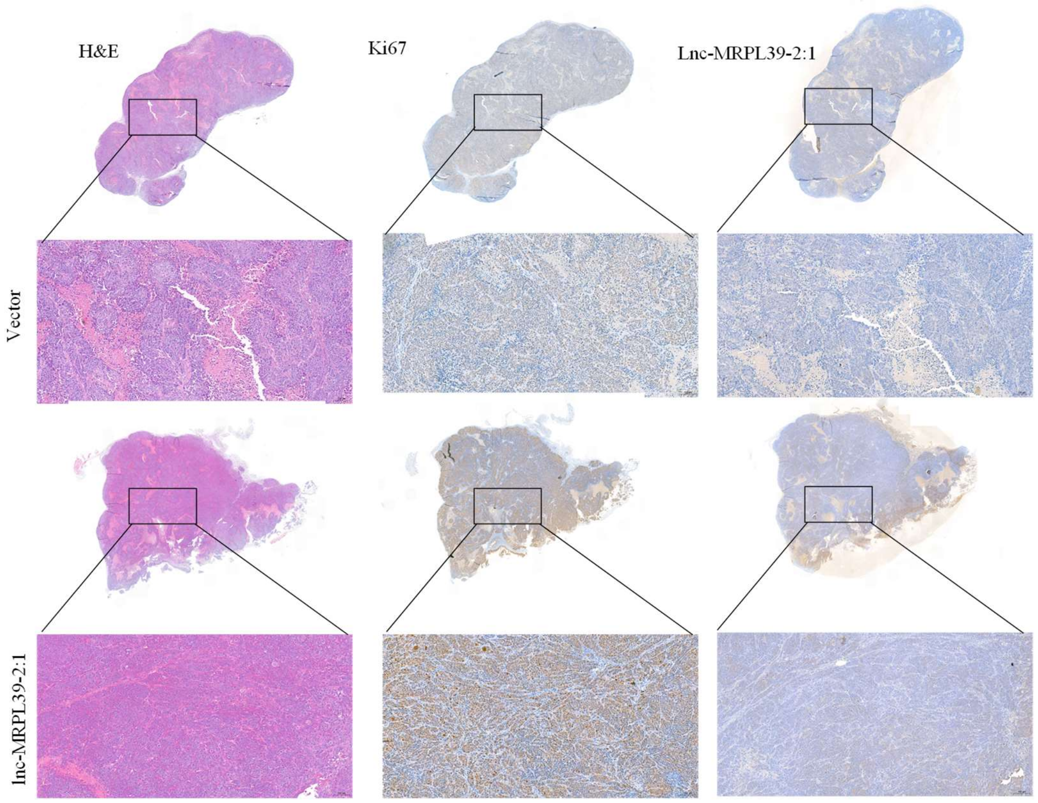

Supplement: Supplementary file 1 — Supplementary figures and tables. [file ijbsv19p2349s1.pdf]
